# Methylglyoxal, a glycolysis metabolite, triggers metastasis through MEK/ERK/SMAD1 pathway activation in breast cancer

**DOI:** 10.1186/s13058-018-1095-7

**Published:** 2019-01-23

**Authors:** Marie-Julie Nokin, Justine Bellier, Florence Durieux, Olivier Peulen, Gilles Rademaker, Maude Gabriel, Christine Monseur, Benoit Charloteaux, Lieven Verbeke, Steven van Laere, Patrick Roncarati, Michael Herfs, Charles Lambert, Jean Scheijen, Casper Schalkwijk, Alain Colige, Jo Caers, Philippe Delvenne, Andrei Turtoi, Vincent Castronovo, Akeila Bellahcène

**Affiliations:** 1 Metastasis Research Laboratory, GIGA-Cancer, University of Liège (ULiège), Pathology Tour, +4 level, Building 23, Avenue Hippocrate 13, 4000 Liège, Belgium; 2 Genomics Platform, GIGA, ULiège, Liège, Belgium; 3 Department of Information Technology, Ghent University, Ghent, Belgium; 4 Translational Cancer Research Unit, University of Antwerp, Antwerp, Belgium; 5 Laboratory of Experimental Pathology, GIGA-Cancer, ULiège, Liège, Belgium; 6 Laboratory of Connective Tissues Biology, GIGA-Cancer, ULiège, Liège, Belgium; 7 Laboratory for Metabolism and Vascular Medicine, Department of Internal Medicine, Maastricht University, Maastricht, The Netherlands; 8 Laboratory of Hematology, GIGA-Inflammation, Infection and Immunity, ULiège, Liège, Belgium; 9 Institut de Recherche en Cancérologie de Montpellier, Inserm U1194, Montpellier, France

**Keywords:** Breast cancer, Methylglyoxal, SMAD1, Metastasis, Carnosine, MAPK

## Abstract

**Background:**

Elevated aerobic glycolysis rate is a biochemical alteration associated with malignant transformation and cancer progression. This metabolic shift unavoidably generates methylglyoxal (MG), a potent inducer of dicarbonyl stress through the formation of advanced glycation end products (AGEs). We have previously shown that the silencing of glyoxalase 1 (GLO1), the main MG detoxifying enzyme, generates endogenous dicarbonyl stress resulting in enhanced growth and metastasis in vivo. However, the molecular mechanisms through which MG stress promotes metastasis development remain to be unveiled.

**Methods:**

In this study, we used RNA sequencing analysis to investigate gene-expression profiling of GLO1-depleted breast cancer cells and we validated the regulated expression of selected genes of interest by RT-qPCR. Using in vitro and in vivo assays, we demonstrated the acquisition of a pro-metastatic phenotype related to dicarbonyl stress in MDA-MB-231, MDA-MB-468 and MCF7 breast cancer cellular models. Hyperactivation of MEK/ERK/SMAD1 pathway was evidenced using western blotting upon endogenous MG stress and exogenous MG treatment conditions. MEK and SMAD1 regulation of MG pro-metastatic signature genes in breast cancer cells was demonstrated by RT-qPCR.

**Results:**

High-throughput transcriptome profiling of GLO1-depleted breast cancer cells highlighted a pro-metastatic signature that establishes novel connections between MG dicarbonyl stress, extracellular matrix (ECM) remodeling by neoplastic cells and enhanced cell migration. Mechanistically, we showed that these metastasis-related processes are functionally linked to MEK/ERK/SMAD1 cascade activation in breast cancer cells. We showed that sustained MEK/ERK activation in GLO1-depleted cells notably occurred through the down-regulation of the expression of dual specificity phosphatases in MG-stressed breast cancer cells. The use of carnosine and aminoguanidine, two potent MG scavengers, reversed MG stress effects in in vitro and in vivo experimental settings.

**Conclusions:**

These results uncover for the first time the key role of MG dicarbonyl stress in the induction of ECM remodeling and the activation of migratory signaling pathways, both in favor of enhanced metastatic dissemination of breast cancer cells. Importantly, the efficient inhibition of mitogen-activated protein kinase (MAPK) signaling using MG scavengers further emphasizes the need to investigate their therapeutic potential across different malignancies.

**Electronic supplementary material:**

The online version of this article (10.1186/s13058-018-1095-7) contains supplementary material, which is available to authorized users.

## Background

A key hallmark of cancer cells is their metabolic reprogramming consisting of enhanced aerobic glycolysis over oxidative respiration. This so called “Warburg effect” leads to the accumulation of methylglyoxal (MG), a highly toxic and reactive dicarbonyl, that spontaneously glycates proteins, nucleic acids and lipids [1]. The reaction of MG with the amino group of proteins leads to the formation of advanced glycation end-products (AGEs) such as hydroimidazolones (MG-Hs) and argpyrimidines [2]. To fight MG, mammalian cells developed a detoxifying system consisting of glyoxalases 1 and 2 (GLO1 and GLO2) that transform MG into D-lactate in the presence of reduced glutathione [3]. The imbalance between MG production and its detoxification results in MG adducts accumulation in cells identified as dicarbonyl stress. The consequences of this cellular stress have been mainly studied in the context of diabetes mellitus and its complications [4]. Collagen, laminin and mitochondrial proteins are examples of MG targets [5]. Because of their slow turnover, long-living proteins such as collagens are more prone to being glycated and cross-linked by MG [6]. Previously, we reported the consistent accumulation of MG protein adducts in patients with breast [7] and colon [8] cancers compared to their respective normal counterparts. Initially, MG was mostly considered a toxic molecule for both normal and cancer cells. Recently, using breast and glioblastoma cell lines, our group has demonstrated that low doses of MG promote tumor growth rather than inhibit it. This hormesis effect of MG seemingly reconciles contrasting data in the literature [9]. In fact, at sub-toxic doses, MG turns out to be beneficial to cancer cells as they acquire resistance to apoptosis and enhanced growth properties.

Furthermore, it remains controversial whether GLO1 in cancer acts as a tumor suppressor or oncogene. Several studies depicted GLO1 as an amplified and/or overexpressed oncogene associated with a poor prognosis in various types of malignant tumors, thus considering the inhibition of GLO1 activity as a potential anti-cancer therapy (for review [10]). Zender and collaborators [11] identified and validated GLO1 as a tumor suppressor gene in hepatocellular carcinoma and knockdown of GLO1 using short hairpin RNAs (shRNAs) increased tumor growth in vivo. In line with these data, we have recently reported that breast cancer cells stably depleted in GLO1 and, consequently, bearing a higher level of MG, exhibit significantly enhanced tumor growth and metastatic potential [12]. We have showed that MG glycates HSP90, which leads to nuclear accumulation of Yes-associated protein (YAP) and the blockade of the Hippo tumor suppressor pathway [12]. If this mechanism has shed some light on the pro-growth effect of MG, it did not provide any clues to explain how this metabolite might enhance the metastatic phenotype of breast cancer cells. Accordingly, the aim of the present study was to ascertain whether MG could specifically have an impact on essential processes and pathways governing migration and metastasis.

Comprehensive RNA-sequencing (RNASeq) analysis of GLO1-depleted cancer cells identified a metastatic transcriptomic signature involving genes tightly associated with cell migration and extracellular matrix (ECM) remodeling, such as collagens and tenascin C. We have demonstrated that MG-induced pro-metastatic signature is linked with the activation of the mitogen-activated protein kinase kinase (MEK)/extracellular signal-related protein kinase (ERK) pathway, which signals through activated SMAD1. We have further demonstrated that MEK/ERK-sustained activation in GLO1-depleted cells notably occurs through the down-regulation of dual specificity phosphate 5 (DUSP5) phosphatase expression upon MG stress in breast cancer cells. Overall, our study demonstrated for the first time that MG stress is able to change the ECM and to regulate migratory signaling pathways, both in favor of enhanced metastatic dissemination of breast cancer cells.

## Methods

### Cell culture and reagents

MDA-MB-231 and MCF7 cancer cell lines were obtained from the American Type Culture Collection (ATCC, Manassas, VA, USA). MDA-MB-468 cells were kindly provided by Dr C. Gilles (University of Liège, Belgium). Cells were cultured in Dulbecco’s modified Eagle’s medium (DMEM) (Lonza) containing 10% fetal bovine serum (FBS, ThermoFisher Scientific) and 2 mM L-glutamine (Lonza). For cell signaling analysis, cells were cultured in DMEM with L-glutamine and without FBS. L-carnosine (C9625), aminoguanidine (396494) and methylglyoxal (MG, M0252) were from Sigma. We excluded the presence of significant formaldehyde contamination (< 3%) in MG (lot #BCBQ9416V) by nuclear magnetic resonance (NMR) analysis. Transforming growth factor (TGF)β (#10021) was from Peprotech. U0126 (S1102) was from Selleckchem. Anti-argpyrimidine antibody (mAb6B) specificity has been previously confirmed by competitive enzyme-linked immunosorbent assay (ELISA) and shown to not react with other MG-arginine adducts [13].

### Extracellular MG quantification

MG measurements were performed as previously described [12]. Briefly, culture medium was collected from GLO1-silenced MDA-MB-231 cells and the corresponding attached cells were counted to normalize MG measurements. Levels of MG were determined by derivatization with O-phenylenediamine (oPD) and analyzed by stable isotope dilution ultra-performance liquid chromatography tandem mass spectrometry (UPLC-MS/MS) as described previously [14].

### In vivo metastatic breast cancer models

All animal experimental procedures were performed according to the Federation of European Laboratory Animal Sciences Associations (FELASA) and were reviewed and approved by the Institutional Animal Care and Ethics Committee of the University of Liège. Animals were housed in the GIGA-accredited animal facility (University of Liège). Eight-week-old female NOD-SCID mice were inoculated via the tail vein with MDA-MB-231 shNT, shGLO1#1 and #2 cells (500000cells/200 μl phosphate-buffered saline (PBS)) and were monitored for 6 weeks for development of metastases. Mice received intra-peritoneal (IP) injection of carnosine (100 mg/kg) three times per week from the day of the engraftment until the end of the experiment (*n* = 12–14 per condition). Lungs were collected and embedded in paraffin for immunohistochemical analysis (IHC).

### Immunohistochemical analysis (IHC)

IHC was performed using a standard protocol as described previously [12] using the following antibodies: anti-vimentin (1:4, clone V9, Ventana Medical Systems), anti-tenascin C (1:1000, ThermoFisher Scientific) and anti-collagen VI α3 (1:200, Sigma). Positive cells were visualized using a 3,3′-diaminobenzidine substrate and the sections were counterstained with hematoxylin. Tissue sections incubated without primary antibody showed no detectable immunoreactivity. Tissue was stained with hematoxylin eosin and Masson's trichrome using standard protocols.

### Evaluation of IHC staining

For vimentin IHC, three different whole immunolabeled tissue sections per lung were digitalized at high resolution (Ventana iScan HT, Ventana Medical Systems). Vimentin-positive metastatic cells were quantified by computerized counts (QuPath 0.1.2 open source software) and verified by manual counting. The number of positive cells to lung area was reported, yielding a count expressed as the number of cells per square millimeter. For tenascin C IHC and Masson’s trichrome staining, a score of 0 was associated with negative staining and a score of 1 with positive staining in tumor cells.

### Plasmids, shRNA and small interfering RNA (siRNA) transfection, quantitative reverse transcription-PCR (qRT-PCR), western blot and immunofluorescence

Standard protocols are detailed in Additional file 1: Supplementary methods.

### Collagen retraction assay

Collagen gels were made as previously described [15]. Briefly, DMEM containing 10% fetal calf serum (FCS) was prepared at the appropriate concentration and at pH 8.3 to accommodate the addition of bovine skin collagen (3 mg/ml final concentration) in HAc 0.1% and MDA-MB-231 (300.000 cells/gel). After polymerization of the collagen, the gels were detached from the walls of the dishes by gentle tapping and swirling. Gel diameter was measured at regular intervals and collagen gel areas were calculated.

### Migration and invasion assays

MDA-MB-231 and MDA-MB-468 cells (2 × 10^5^ cells) were suspended in serum-free DMEM medium (0.1% bovine serum albumin (BSA), 1% penicillin/streptomycin (pen/strep)) and seeded into the upper part of Transwell filters (pore size 8 μm, Costar) for migration or Transwell filters precoated with Matrigel for invasion (BD Biosciences). The lower compartment was filled with DMEM containing 1% BSA, 1% pen/strep and 10% FBS. After 16 h or 24 h incubation at 37 °C for migration and invasion, respectively, migrating cells were stained with Diff-Quick (Medion Diagnostics). Inserts were scanned at 10× magnification (Eclipse Ti, Nikon). The area covered by migrating/invading cells was quantified by densitometry (ImageJ). Data are expressed as relative migration/invasion ability compared to control cells.

### Extracellular flux analyzes

MCF7 (15,000 cells/well) and MCF7-M (60,000 cells/well) cells were seeded in Seahorse XFp mini-plates (Agilent) and analyzed using the Seahorse glycolysis stress test according to manufacturer’s recommendations. Cells were first challenged with glucose (10 mM) then successively stressed with oligomycin (1 μM) and 2-deoxyglucose (50 mM). All results were normalized to protein quantification.

### Glucose uptake

Cells were incubated in glucose-free medium in the presence of 2-NBDG (ThermoFisher Scientific) for 30 min and further analyzed by flow cytometry (BD Biosciences). Median fluorescence intensity (MFI) was calculated.

### GLO1 activity assay

GLO1 activity was assessed as previously described [7]. Briefly, proteins were extracted with radioimmunoprecipitation (RIPA) buffer, quantified and mixed with a pre-incubated (15 min at 25 °C) equimolar (1 mM) mixture of MG and GSH (Sigma) in 50 mM sodium phosphate buffer, pH 6.8. S-D-lactoylglutathione formation was followed spectrophotometrically by the increase in absorbance at 240 nm at 25 °C. GLO1 activity data are expressed as arbitrary units (A.U.) of enzyme per milligram of proteins.

### Statistical analysis

Data from two groups were compared using the unpaired Student’s *t* test with or without Welsch’s correction according to homoscedasticity. Experimental data from more than two groups were compared using one-way or two-way analysis of variance (ANOVA) depending on the number of grouping factors. Dunnett’s test was applied for simple comparisons while Student-Newman-Keul’s (one-way ANOVA) or Bonferroni’s (two-way ANOVA) tests were used for multiple comparisons. In the case of discrete variables (IHC scores) or non-normally distributed variables, groups were compared using Mann-Whitney’s U test. Outliers were detected using whisker box plots. A bilateral *p* value <0.05 was considered statistically significant with a 95% confidence interval.

## Results

### RNASeq analysis of GLO1-depleted MDA-MB-231 breast cancer cells highlights a pro-metastatic signature

**Fig. 1:**
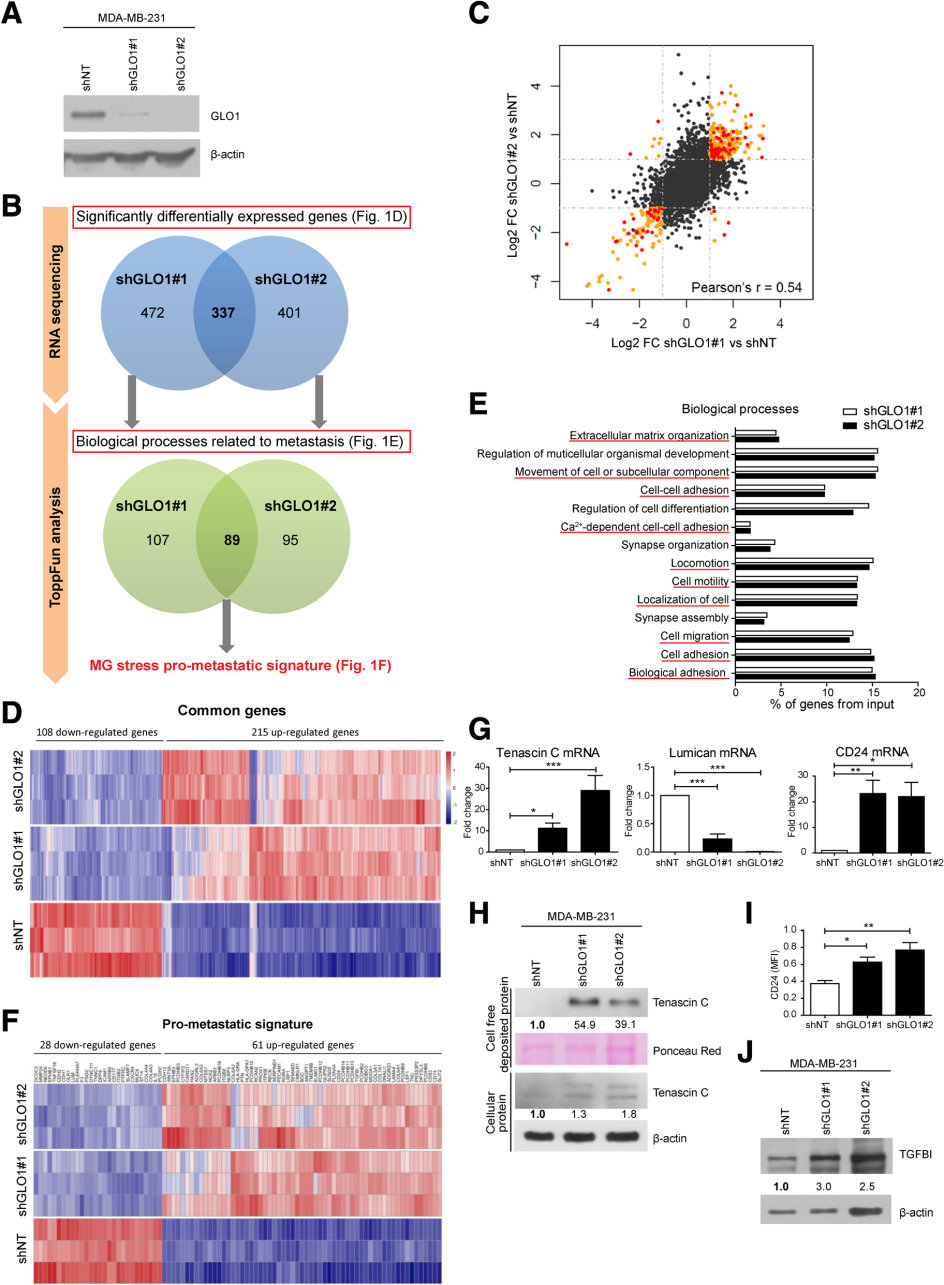
RNA sequencing analysis of glyoxalase 1 (GLO1)-depleted MDA-MB-231 cells highlights a pro-metastatic signature related to methylglyoxal (MG) stress. **a** GLO1 protein level in shNT, shGLO1#1 and #2 MDA-MB-231 cells. β-actin protein is used as loading control. Western blot is representative of three independent experiments. **b** Different steps of the high-throughput transcriptome analysis of GLO1-depleted MDA-MB-231 cells. **c** Correlation analysis of messenger RNA (Mrna) expression changes measured upon GLO1 depletion in shGLO1 (#1 and #2) clones when compared to shNT clone. Orange and red dots represent genes that are significantly differentially expressed (*q* < 0.05 and log2 fold change (FC) > 1). Red dots represent genes of the pro-metastatic signature. **d** Heatmap representing gene expression levels in the three replicates of all three conditions (shNT, shGLO1#1 and shGLO1#2 MDA-MB-231 cells) for genes significantly differentially expressed for both shGLO1 clones. Color scale corresponds to the expression *Z*-score across all samples calculated for each gene. **e** Significantly differentially expressed genes were analyzed for their gene ontology using ToppFun Suite software. Graph represents the percentage of genes from the input in the 12 most significantly affected biological processes for both shGLO1#1 and shGLO1#2 cells. Biological processes underlined in red are involved in the metastatic cascade. **f** All the genes listed in the biological processes related to metastasis were defined as the pro-metastatic gene signature of GLO1-depleted MDA-MB-231 cells. Down-regulated and up-regulated genes of the pro-metastatic gene signature are represented in a heatmap. Color scale corresponds to *Z*-score. **g** Tenascin C, Lumican and CD24 mRNA levels were assessed by qRT-PCR in GLO1-depleted MDA-MB-231 cells. **h** Tenascin C protein expression was detected in both cell-free deposited protein extracts and total cellular proteins of shGLO1 cells. **i** Cell surface CD24 protein level was assessed by flow cytometry. **j** TGFBI protein expression in GLO1-depleted MDA-MB-231 cells was assessed by western blot. All immunoblot data were quantified by densitometric analysis and normalized for ponceau red or β-actin. Numbers represent fold increase relative to the condition shown with bold number. Western blots are representative of two independent experiments. Data were analyzed using one-way analysis of variance followed by Dunnett post-hoc test and shown as mean values ± SEM of three independent experiments. *p < 0.05, **p < 0.01 and ***p < 0.001

In an attempt to unveil the mechanisms by which MG stress enhances the metastatic potential of breast cancer cells, we performed genome-wide messenger RNA (mRNA) profiling of MDA-MB-231 GLO1-depleted cells. We first validated GLO1 depletion by western blot and the subsequent increased MG production in stably GLO1-silenced MDA-MB-231 cells (Fig. 1a and Additional file 2: Figure S1A). Using a workflow consisting of RNASeq followed by ToppFun analysis (Fig. 1b), we have identified 809 and 738 genes that were significantly differentially expressed in shGLO1#1 and #2, respectively, when compared to control cells. ShGLO1#1 and #2 clones had consistent gene modulation and overall good correlation (Fig. 1c and Additional file 2: Figure S1B). Among differentially expressed genes, 337 genes were common to both shGLO1 clones, among which 323 genes were consistently up-regulated or down-regulated (Fig. 1d). Based on Gene Ontology analysis, differentially expressed genes were associated with key steps in the metastatic cascade including cell adhesion, migration and ECM reorganization (Fig. 1e). Globally, 196 and 184 significantly modulated genes were linked to migration and adhesion processes in shGLO1#1 and #2, respectively. The combination of these two sets of genes resulted in a unique dicarbonyl stress signature consisting of 296 genes related to the pro-metastatic behavior of breast cancer cells, with 89 differentially expressed genes common between the two shGLO1 clones (Fig. 1f).

### MG-stress-related pro-metastatic signature highlights the regulation of major ECM proteins by breast cancer cells

**Fig. 2:**
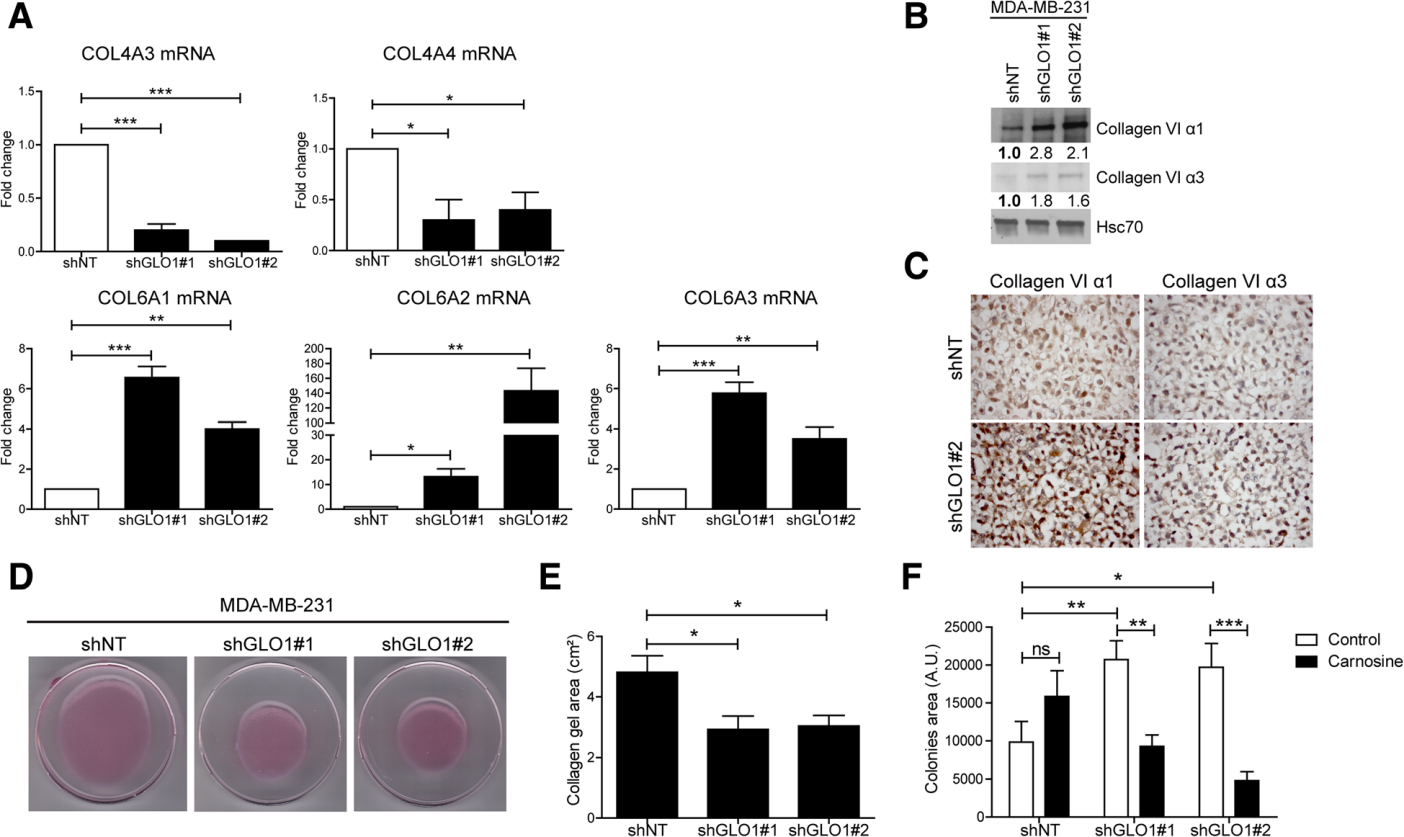
Glyoxalase 1 (GLO1) depletion modifies the expression of extracellular matrix (ECM) proteins by breast cancer cells and promotes collagen gel retraction and anchorage-independent growth. **a** Collagen (COL)4A3, COL4A4, COL6A1, COL6A2 and COL6A3 messenger RNA (mRNA) levels were assessed by qRT-PCR in GLO1-depleted MDA-MB-231 cells. Data were analyzed using one-way analysis of variance (ANOVA) followed by Dunnett post-hoc test and shown as mean values ± SEM of three independent experiments. **b** Collagen VI α1 and α3 protein levels in MDA-MB-231 shGLO1 cells. Immunoblot data were quantified by densitometric analysis and normalized to Hsc70. Numbers represent fold increase relatively to the condition shown with bold number. Western blots are representative of two independent experiments. **c** Collagen VI α1 and α3 protein levels in MDA-MB-231 shGLO1#2 and control (shNT) cells were assessed by immunohistochemical analysis (IHC). **d** Representative collagen gel retraction assays performed with MDA-MB-231 shGLO1 cells. **e** Quantification of collagen gel area after 6 days. Data were analyzed using one-way ANOVA followed by Dunnett post-hoc test and shown as mean values ± SEM of five independent experiments. **f** Quantification of colonies formed in a soft agar matrix by GLO1-silenced MDA-MB-231 cells treated with carnosine. Data were analyzed using two-way ANOVA followed by Bonferroni post-hoc test and shown as mean values ± SEM of three independent experiments. ns, not significant; *p < 0.05, **p < 0.01 and ***p < 0.001

We validated the expression level of remarkable up-regulated and down-regulated genes known to be linked to the metastatic potential of cancer cells (Additional file 2: Table S1). Using RT-qPCR, tenascin C and CD24 mRNA levels proved to be significantly overexpressed in GLO1-depleted cells (Fig. 1g). Lumican, a small leucine-rich proteoglycan the down-regulation of which has been associated with lung invasion in different cancer types, was significantly inhibited at the mRNA level upon MG stress (Fig. 1g). In order to demonstrate that MG stress directly regulates the expression of these genes, we treated MDA-MB-231 cells with MG. Similar to GLO1 silencing, MG treatment significantly increased tenascin C and CD24 mRNA levels and decreased lumican mRNA level (Additional file 2: Figure S1C). At the protein level, tenascin C overexpression was evidenced in both total protein extracts and secreted proteins deposited by cultured GLO1-depleted cells (Fig. 1h) while increased CD24 cell surface expression was confirmed using fluorescence-activated cell sorting (FACS) analysis (Fig. 1i). TGFBI, an RGD-containing ECM protein that binds to collagens, was also increased in GLO1-silenced MDA-MB-231 cells (Fig. 1j). The decrease in type IV collagens (COL4A3 and COL4A4) associated with an increase in type VI collagens (COL6A1, COL6A2 and COL6A3) particularly attracted our attention (Additional file 2: Table S1). Indeed, this inverse regulation has been linked to cancer progression and metastasis [16]. We validated this specific modulation of collagen gene expression in cancer cells upon GLO1 depletion at both mRNA (Fig. 2a) and protein (Fig. 2b and c) levels. To the best of our knowledge, this is the first evidence of the contribution of endogenous MG stress to ECM composition directly by breast cancer cells.

### GLO1-depleted cancer cells show increased collagen gel retraction and anchorage-independent growth

The well-established role of ECM remodeling in structuring a microenvironment favoring cancer cell progression prompted us to explore the behavior of high MG stress cancer cells using the collagen gel retraction assay, an in vitro model well-suited to study cell-mediated reorganization of the ECM. Accordingly, we analyzed the capacity of GLO1-silenced MDA-MB-231 cells to compact collagen fibers in the presence of 10% FCS. After 6 days, GLO1 deficient cells induced significantly higher collagen gel retraction than control cells (Fig. 2d and e). Anchorage-independent growth ability of breast cancer cells correlates with their metastatic ability [17]. In anchorage-independent assays, GLO1-silenced MDA-MB-231 cells formed significantly more colonies than control cells and this difference was abrogated by the natural MG scavenger carnosine (Fig. 2f and Additional file 2: Figure S2).

### GLO1-depleted breast cancer cells efficiently colonize the lung in an experimental metastatic model in vivo and carnosine has an inhibitory effect

**Fig. 3:**
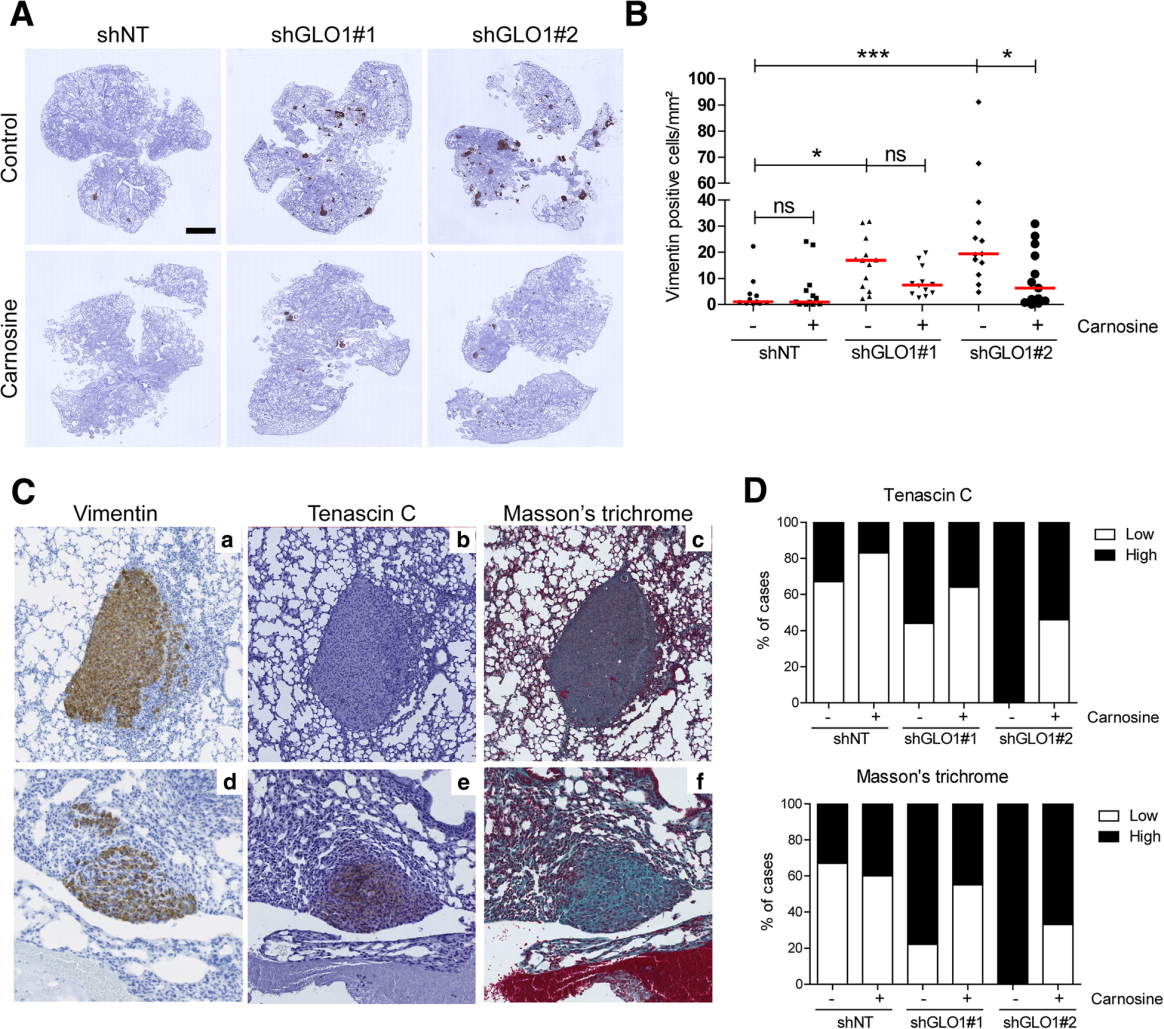
Glyoxalase 1 (GLO1)-depleted breast cancer cell efficiently colonize the lung in an experimental metastatic model in vivo and inhibitory effect of carnosine. **a** MDA-MB-231 shGLO1#1 and #2 and control shNT cells were injected into the tail vein of NOD-SCID mice (12–14 mice/group). Mice were treated with carnosine by intraperitoneal injection (100 mg/kg, 3 times/week). After 6 weeks, mice were sacrificed and lungs were collected. Representative human vimentin immunohistochemical analysis (IHC) of whole lungs shows metastatic tumor lesions. Bar represents 2 mm. **b** Quantification of vimentin-positive cells on three representative whole lung sections per mouse. Each dot represents one case and red bars represent median. Data were analyzed using one-way analysis of variance. **c** Human vimentin (a, d) and tenascin C (b, e) IHC and Masson’s trichrome staining (c, f) were performed on whole lungs from mice injected into the tail vein with MDA-MB-231 shGLO1 cells. Representative stains are shown for tenascin C and Masson’s trichrome scored as low (b and c, respectively) or high (e and f, respectively). Magnification 20×. **d** Quantification of tenascin C and Masson’s trichrome staining on lung sections from mice injected with GLO1-silenced MDA-MB-231 cells treated with carnosine. ns, not significant; *p < 0.05 and ***p < 0.001

To test the relevance of the pro-metastatic MG stress signature in vivo*,* we decided to use the tail vein injection mouse model that recapitulates the major steps of the metastatic cascade (migration/invasion, proliferation and survival) independently from the growth of the primary tumor. We observed that GLO1-depleted cells injected into the tail vein of NOD-SCID mice induced a significant increase in pulmonary tumor burden when compared with control (Fig. 3a). In the same model, carnosine intra-peritoneal administration significantly reduced lung colonization thus connecting this aggressive characteristic with MG stress (Fig. 3a and b). Finally, IHC for tenascin C and collagen deposition assessed by Masson’s trichrome staining in metastatic lung sections showed high detectable levels of both ECM components (Fig. 3c and d), which were consistently lower in metastatic foci of carnosine-treated mice (Fig. 3d). Next, we examined whether enhanced anchorage-independence growth and metastatic potential (i.e., lung colonization) of GLO1-depleted cells correlated with increased invasion and migration ability in vitro.

### MG stress increases invasive and migratory abilities of breast cancer cells in vitro

**Fig. 4:**
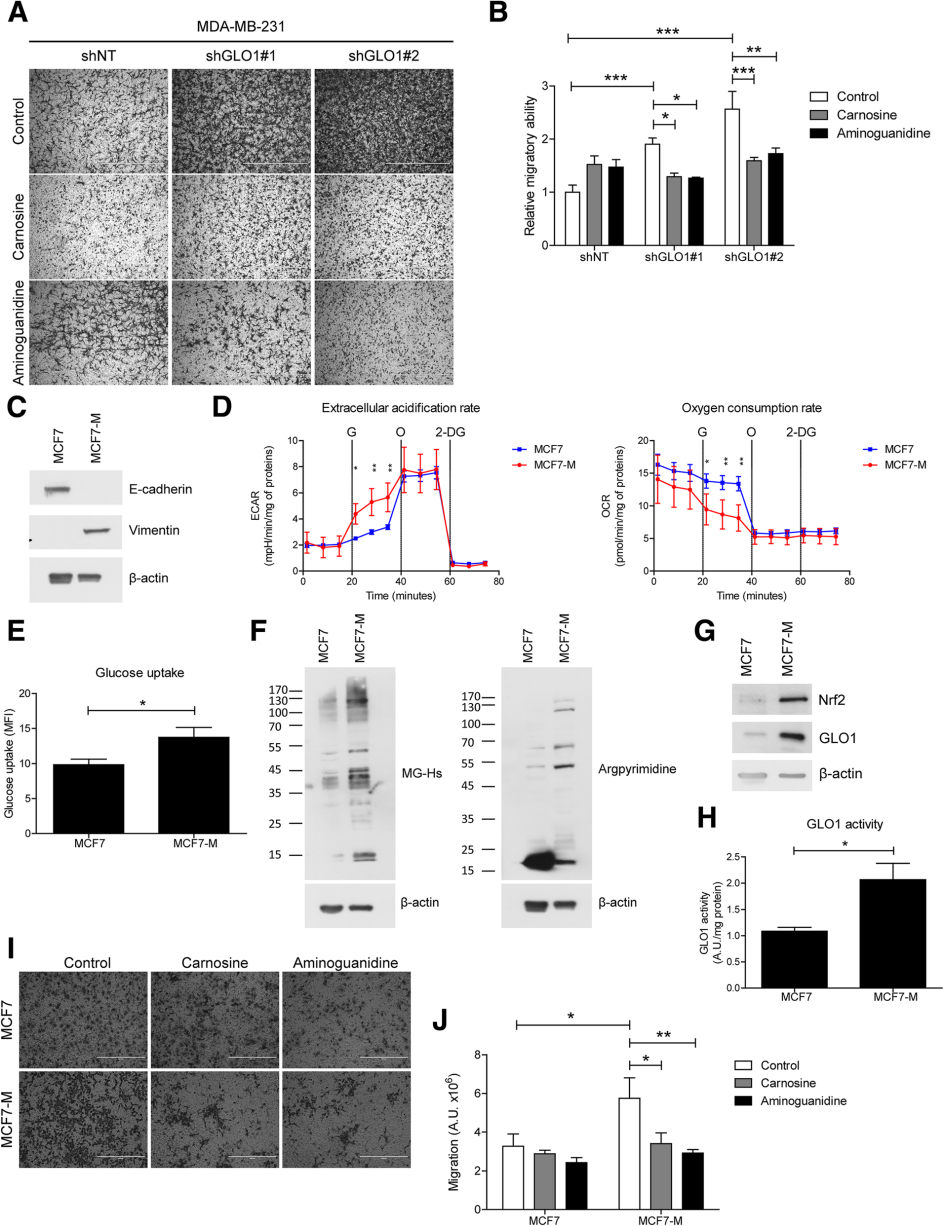
Dicarbonyl stress promotes migration and invasion of breast cancer cells. **a** Migration ability of glyoxalase 1 (GLO1)-depleted MDA-MB-231 toward serum was assessed using Transwell filters. Where indicated, cells were pre-treated with the methylglyoxal (MG) scavengers, carnosine and aminoguanidine, 24 h prior to the assay. Representative filters are shown for each condition. Scale bar represents 400 μm. **b** Quantification of migratory ability of GLO1-silenced MDA-MB-231 cells treated with carnosine and aminoguanidine. Data were analyzed using two-way analysis of variance (ANOVA) followed by Bonferroni post-hoc test and shown as mean values ± SEM of three independent experiments. **c** E-cadherin and vimentin expression in MCF7 and MCF7-M cells. β-actin protein level was used as loading control. A representative western blot of two independent experiments is shown. **d** Extracellular acidification (ECAR) and oxygen consumption (OCR) rates in MCF7 and MCF7-M cells using Seahorse flux analyzer. G, O and 2-DG correspond to injection of glucose, oligomycin and 2-deoxyglucose, respectively. Data were analyzed using two-way ANOVA and are representative of two independent experiments. **e** Glucose uptake was assessed in MCF7 and MCF7-M cells using FACS analysis. Data were analyzed using Student’s *t* test and are shown as mean values ± SEM from three independent experiments. **f** MG-Hs and argpyrimidine MG adducts levels were detected by immunoblot using specific antibodies in MCF7 and MCF7-M cells, with β-actin as loading control. **g** GLO1 and Nrf2 expression in MCF7 and MCF7-M cells. β-actin protein is used as loading control. Western blot is representative of three independent experiments. **h** GLO1 maximal activity was measured in MCF7 and MCF7-M cells and expressed as arbitrary units (A.U.) per mg of proteins. Data were analyzed using Student’s *t* test and are shown as mean values ± SEM of three independent experiments. **i** Migration ability toward serum of MCF7 and MCF7-M cells was assessed using Transwell filter. Cells were pre-treated with carnosine and aminoguanidine MG scavengers for 24 h prior to the assay. Representative filters are shown for each condition. Scale bar represents 400 μm. **j** Quantification of MCF7 and MCF7-M cells migration assays. Data were analyzed using two-way ANOVA followed by Bonferroni post-hoc test and are shown as mean values ± SEM of three independent experiments. *p < 0.05, **p < 0.01 and ***p < 0.001

The migratory ability of GLO1-depleted cancer cells increased up to 2.5-fold compared with control cells (Fig. 4a and b). This increased migratory ability was reversed by overexpressing GLO1 in shGLO1#1 MDA-MB-231 cells (Additional file 2: Figure S3A and B). GLO1 rescue was evaluated by western blot (Additional file 2: Figure S3C). Additionally, the pre-treatment of GLO1-depleted cells with carnosine and aminoguanidine, MG scavengers, decreased their migration to control level (Fig. 4a and b). GLO1 depletion was also significantly associated with augmented invasive potential in vitro, which was efficiently reversed by pre-treatment with both scavengers (Additional file 2: Figure S3D and E). To exclude the possibility of this MG-induced effect being anecdotal to MDA-MB-231 cells, we exploited a model of highly tumorigenic and motile MCF7-M breast cancer cells, generated by prolonged mammosphere culture. In good accordance with previous reports [18, 19], MCF7-M cells exhibited a stable mesenchymal phenotype (Fig. 4c and Additional file 2: Figure S4A), enhanced glycolysis (Fig. 4d and Additional file 2: Figure S4B) and glucose uptake (Fig. 4e). We first demonstrated that MCF7-M cells were associated with significantly higher accumulation of MG-Hs and argpyrimidine adducts than in the control parental MCF7 cells (Fig. 4f). These data are consistent with our recent demonstration that glycolytic cancer cells are more prone to control MG toxicity than less glycolytic cancer cells, notably through the expression of elevated levels of both GLO1 and its cognate transcriptional regulator Nrf2 [9]. Accordingly, MCF7-M had a high GLO1 protein level and activity and elevated Nrf2 expression when compared with MCF7 cells (Fig. 4g and h). Finally, on treatment with carnosine and aminoguanidine, the migratory capacity of MCF7-M cells significantly was reverted to the level of parental cells (Fig. 4i and j), indicating the implication of MG stress in this process. These data, generated using two experimental cancer models, support the conclusion that MG dicarbonyl stress promotes the migration and invasion of breast cancer cells. We selected cell migration ability to be used all along the following mechanistic exploration as a functional readout of MG stress in breast cancer cells.

### Both endogenous and exogenous MG stress are associated with (hyper)activation of the MEK/ERK signaling pathway in breast cancer cells that is linked to their enhanced migratory potential

**Fig. 5:**
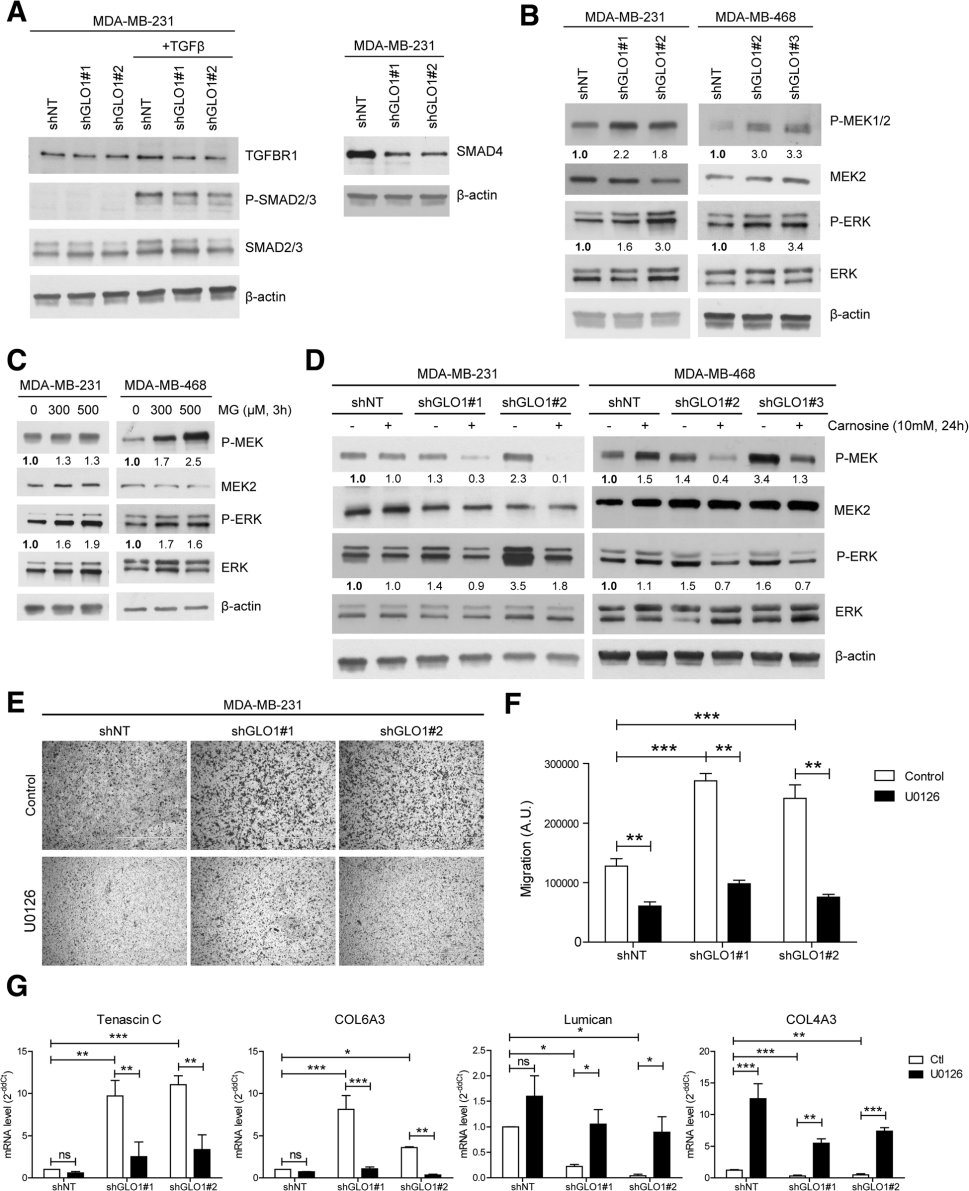
Methylglyoxal (MG) stress induces (hyper)activation of the mitogen-activated protein kinase (MAPK) signaling pathway in breast cancer cells. **a** Transforming growth factor (TGF)βR1, P-SMAD2/3 and SMAD4 protein levels in glyoxalase 1 (GLO1)-depleted MDA-MB-231 cells. Cells were treated with TGFβ 2.5 ng/ml for 2 h where indicated. **b** P-MEK1/2, MEK2, P-ERK and ERK expression in GLO1-silenced MDA-MB-231 and MDA-MB-468 cells cultured without serum for 24 h. **c** P-MEK1/2, MEK2, P-ERK and ERK protein levels in MDA-MB-231 and MDA-MB-468 cells treated with MG at the indicated concentrations for 3h in serum-free medium. **d** P-MEK1/2, MEK2, P-ERK and ERK expression in GLO1-silenced MDA-MB-231 and MDA-MB-468 cells treated with carnosine for 24 h in serum-free medium. All immunoblots were quantified by densitometric analysis and normalized for β-actin. Numbers represent fold increase relative to the condition shown with bold number. Western blot is representative of three independent experiments. **e** Migration ability toward serum of MDA-MB-231 shGLO1 cells pre-treated with MEK inhibitor, U0126 (10 μM, 3 h), was assessed using Transwell filters. Representative filters are shown for each condition. Scale bar represents 400 μm. **f** Quantification of migration assays of GLO1-silenced MDA-MB-231 cells treated with U0126. **g** Tenascin C, COL6A3, lumican and COL4A3 messenger RNA (mRNA) levels in GLO1-depleted MDA-MB-231 cells treated with U0126 for 3 h in serum-free medium were assessed by qRT-PCR. Data were analyzed using two-way analysis of variance followed by Bonferroni post-hoc test and are shown as mean values ± SEM of three independent experiments. *p < 0.05, **p < 0.01 and ***p < 0.001

As a major component of the tumor microenvironment, the ECM regulates many pathways in cancer cells, including TGF-β/bone morphogenic protein (BMP), PI3K/AKT, ERK, c-Jun N terminal kinase (JNK), Src-focal adhesion kinase (FAK) and Rho-GTPases [20]. Following the rationale that TGFβ is a key regulator of ECM synthesis in cancer, we first explored the impact of MG stress on the TGFβ pathway. Under basal conditions, neither GLO1-depleted nor control MDA-MB-231 cells showed detectable phosphorylated SMAD2/3, albeit, they were responsive to TGFβ as indicated by SMAD2/3 activation upon TGFβ stimulus (Fig. 5a). Interestingly, SMAD4 expression was significantly decreased in GLO1-depleted cells, suggesting that the canonical TGFβ pathway was dispensable upon MG stress induction in these cells (Fig. 5a).

We therefore explored alternative signaling pathways downstream of tyrosine kinase receptors and found that the level of phosphorylated MEK and ERK was increased in GLO1-depleted MDA-MB-231 cells (Fig. 5b). Knowing that MDA-MB-231 cells are mutated for KRAS and have a constitutive basal activation of MEK/ERK signaling pathway, we postulated that GLO1 silencing might be associated with the specific hyperactivation of this pathway. To verify this hypothesis, we next attempted to recapitulate this MG effect on MEK/ERK activity in MDA-MB-468 cells, a triple-negative breast cancer cell line that is wild-type for KRAS. Interestingly, MDA-MB-468 clones stably depleted for GLO1 (Additional file 2: Figure S5A) demonstrated a significant increase in MEK/ERK activation over the basal levels of control cells (Fig. 5b). Similar to MDA-MB-231 cells, we found that CD24 and lumican, two genes from the pro-metastatic signature, were increased and decreased, respectively, in GLO1-depleted MDA-MB-468 cells (Additional file 2: Figure S5B). These changes were also recapitulated upon MG treatment in control MDA-MB-468 cells (Additional file 2: Figure S5C). We were also able to recapitulate both enhanced migratory (Additional file 2: Figure S5D and E) and anchorage-independent growth (Additional file 2: Figure S5F and G) abilities in GLO1-depleted MDA-MB-468 cells that both consistently reverted on treatment with carnosine.

To consolidate the existence of a link between MG stress and MEK/ERK activation, we cultured MDA-MB-231 and MDA-MB-468 cells in the presence of increasing concentrations of exogenous MG. MG treatment induced P-MEK and P-ERK in both cell lines (Fig. 5c). Along the same line, carnosine prevented MEK/ERK activation in both shGLO1 MDA-MB-231 and MDA-MB-468 cells (Fig. 5d). Based on these data, we sought to corroborate the functional relevance of MG-mediated MEK/ERK hyperactivation with the enhanced migratory ability of GLO1-depleted breast cancer cells. Therefore, we treated GLO1-depleted MDA-MB-231 cells with U0126 MEK inhibitor. U0126 significantly prevented the increased migratory capacity observed for breast cancer cells under MG stress (Fig. 5e and f). Supporting this observation further, the expression of representative genes from the MG stress pro-metastatic signature, which were either induced (tenascin C, COL6A3) or repressed (lumican and COL4A3), reverted consistently in the presence of U0126 at the mRNA level in MDA-MB-231 cells (Fig. 5g). The data gathered so far demonstrate that MG dicarbonyl stress induces (hyper)activation of MEK/ERK signalization, independently of KRAS status, which sustains the regulation of pro-metastatic gene expression and migratory capacity of breast cancer cells.

### (Hyper)activation of the MEK/ERK pathway leads to SMAD1 phosphorylation and regulation of MG stress pro-metastatic signature genes

**Fig. 6:**
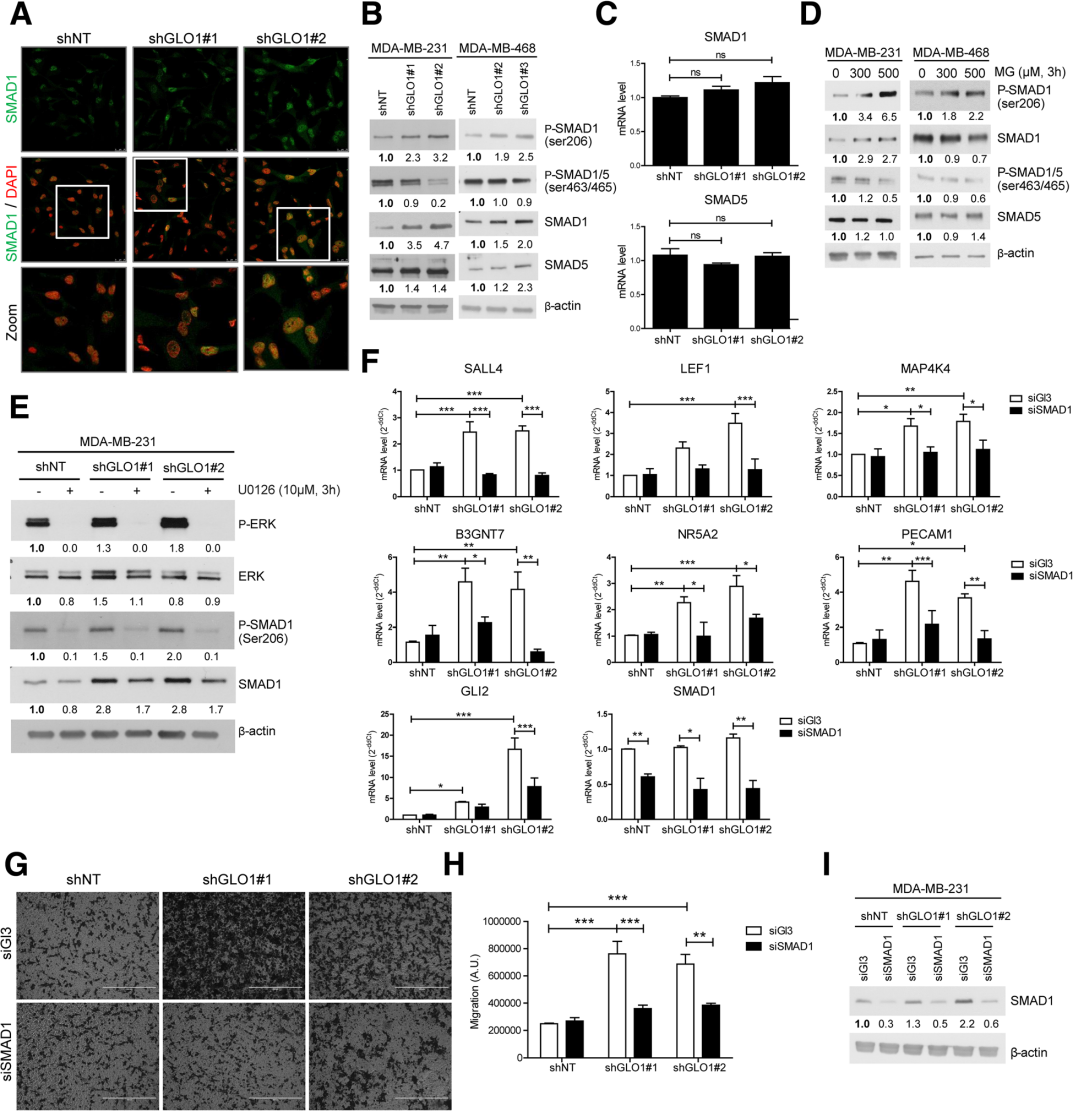
(Hyper)activation of mitogen-activated protein kinase kinase (MEK) signalization leads to SMAD1 phosphorylation and regulation of methylglyoxal (MG) stress pro-metastatic signature genes in breast cancer cells. **a** SMAD1 immunofluorescence staining in glyoxalase 1 (GLO1)-silenced MDA-MB-231 cells cultured in serum-free conditions. Data are representative of three independent experiments. Magnification × 630. Zoomed pictures (white square) are shown where indicated. **b** P-SMAD1 (ser206), P-SMAD1/5 (ser463/465), SMAD1 and SMAD5 protein levels in GLO1-silenced MDA-MB-231 and MDA-MB-468 cells cultured without serum for 24 h. **c** SMAD1 and SMAD5 mRNA levels in GLO1-depleted MDA-MB-231 cells were assessed by qRT-PCR. Data were analyzed using one-way analysis of variance (ANOVA) followed by Dunnett post-hoc test and shown as mean values ± SEM of three independent experiments. **d** P-SMAD1 (ser206), P-SMAD1/5 (ser463/465), SMAD1 and SMAD5 protein level in GLO1-silenced MDA-MB-231 and MDA-MB-468 cells treated with MG at the indicated concentrations for 3 h in serum-free medium. **e** (P-)ERK and (P-)SMAD1 (ser206) protein level in GLO1-silenced MDA-MB-231 cells treated with U0126 MEK inhibitor (10 μM, 3 h) in serum-free medium. **f** SMAD1 target genes mRNA levels were assessed using qRT-PCR in SMAD1-silenced MDA-MB-231 shGLO1 cells. SMAD1 mRNA level was assessed to validate efficient SMAD1 siRNA silencing. **g** Migration ability toward serum of SMAD1-silenced MDA-MB-231 shGLO1 cells was assessed using Transwell filters. Representative filters are shown for each condition. Scale bar represents 400 μm. **h** Quantification of migration assays of SMAD1-silenced MDA-MB-231 shGLO1 cells. **i** SMAD1 protein level assessed by immunoblot to validate SMAD1-silencing using siRNAs in GLO1-depleted MDA-MB-231 cells (related to panels **g** and **h**). Immunoblot data were quantified by densitometric analysis and normalized for β-actin. Numbers represent fold increase relative to the condition shown with bold number. All western blots are representative of three independent experiments. Data were analyzed using two-way ANOVA followed by Bonferroni post-hoc test and shown as mean values ± SEM of three independent experiments. *p < 0.05, **p < 0.01 and ***p < 0.001

To understand how (hyper)activation of the MEK/ERK signaling cascade in GLO1-depleted breast cancer cells relates to their metastatic potential, we first considered that MEK/ERK signaling could be mediated by the physical translocation of ERK in the nucleus. Using immunofluorescence, we excluded this possibility as GLO1-silenced cells showed P-ERK levels that were comparable to control cells using both MDA-MB-231 and MDA-MB-468 cells (Additional file 2: Figure S6A and B, respectively). Early studies involving expression of epitope-tagged SMAD1 indicated that it was generally involved in BMP, but not in TGFβ signaling [21, 22]. More recent data demonstrate that activated Ras/MEK pathway could participate in SMAD1 nuclear transcriptional activity [23, 24]. Based on that, we next evaluated SMAD1 expression and localization in GLO1-depleted MDA-MB-231 cells. As shown in representative immunofluorescence pictures, higher SMAD1 levels were detectable in the nucleus of GLO1-silenced MDA-MB-231 cells than in control cells (Fig. 6a). Phosphorylation of SMAD1 occurs at serine 206 (Ser 206) and serine 463/465 (Ser 463/465) following the activation of MAPK and BMP pathways, respectively [25]. In GLO1-depleted MDA-MB-231 and MDA-MB-468 cells (Fig. 6b), we evidenced a specific increase in P-SMAD1 (Ser 206) and a decrease in P-SMAD1/5 (Ser 463/465) that effectively points to cross-talk with MAPK signaling. We observed no significant change in SMAD1 and SMAD5 mRNA levels (Fig. 6c). GLO1 rescue in shGLO1#1 MDA-MB-231 cells reduced P-SMAD1 (Ser 206) (Additional file 2: Figure S7A). Consistently, MDA-MB-231 and MDA-MB-468 cells cultured in the presence of MG showed significant induction of total SMAD1 and P-SMAD1 (Ser 206) and decreased (Ser 463/465) (Fig. 6d). U0126 treatment further demonstrated the link with the MEK cascade as it strongly reduced the level of P-SMAD1 (Ser 206) in MDA-MB-231 cells (Fig. 6e). MDA-MB-468 cells, which are considered as high SMAD1 expressers when compared with other breast cancer cell lines [26], showed a similar pattern of SMAD1 serine phosphorylation upon MG treatment (Fig. 6d). Metastatic MCF7-M cells, bearing high endogenous MG stress, showed elevated total and phosphorylated SMAD1 levels when compared with parental MCF7 cells (Additional file 2: Figure S7B). SMAD1 inhibition (Additional file 2: Figure S7E) significantly decreased the migratory potential of MCF7-M cells (Additional file 2: Figure S7C and D).

Next, we further explored this new link between SMAD1 effector activation and MG stress signature. To this end, we evaluated the impact of GLO1 depletion on the expression of MG stress signature genes known to be SMAD1 targets. Seven out of 11 SMAD1 target genes, shown to be up-regulated under shGLO1 condition in RNASeq analysis, were significantly reduced upon SMAD1 silencing in GLO1-depleted MDA-MB-231 (Fig. 6f). The same experiment conducted on GLO1-depleted MDA-MB-468 cells revealed 5 out of 11 MG stress signature genes regulated by SMAD1, amongst which the PECAM1, GLI2 and B3GNT7 genes were common with MDA-MB-231 evaluation (Additional file 2: Figure S8A). Efficient silencing of SMAD1 was validated at the mRNA level in MDA-MB-231 (Fig. 6f) and in MDA-MB-468 cells (Additional file 2: Figure S8A). The silencing of SMAD1 in both GLO1-depleted cellular models had an impact on their migration ability and brought it back to control cell levels (Fig. 6g and h and Additional file 2: Figure S8B and C), as shown above for MCF7-M cells (Additional file 2: Figure S7B and C). Efficient silencing of SMAD1 was validated at the protein level (Fig. 6i and Additional file 2: Figure S8D). These results indicated that MG dicarbonyl stress induced the MEK/ERK/SMAD1 cascade (hyper)activation that controls the expression of pro-metastatic genes and impacts on migration in breast cancer cells.

### MG stress is associated with a global decrease in phosphatase gene expression in breast cancer cells

**Fig. 7:**
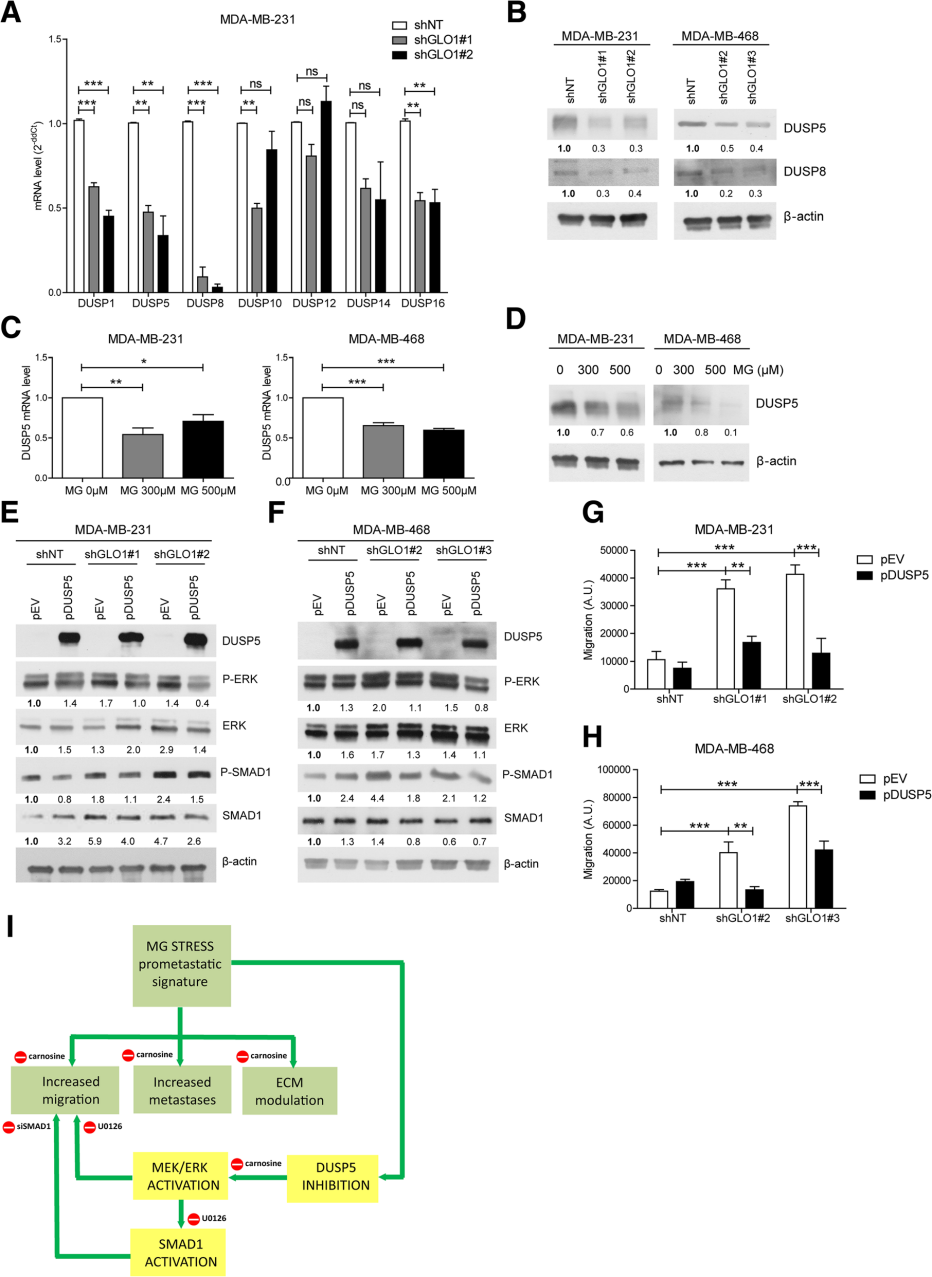
Methylglyoxal (MG)-related invasive phenotype is associated with a decrease in DUSP5 phosphatase expression in breast cancer cells. **a** Dual specificity phosphate (DUSP)1, 5, 8, 10, 12, 14 and 16 phosphatase messenger RNA (mRNA) levels in glyoxalase 1 (GLO1)-depleted MDA-MB-231 cells were quantified by qRT-PCR. **b** DUSP5 and DUSP8 protein expression in MDA-MB-231 and MDA-MB-468 shGLO1 cells cultured in serum-free medium for 24 h was assessed using immunoblot. DUSP5 mRNA (**c**) and protein (**d**) levels in MDA-MB-231 and MDA-MB-468 cells, respectively, treated with MG at the indicated concentrations for 3 h in serum-free medium. **e** and **f** DUSP5, (P-)extracellular signal-related protein kinase (ERK) and (P-)SMAD1 (ser206) protein levels were assessed using immunoblot in GLO1-silenced MDA-MB-231 and MDA-MB-468 cells upon DUSP5 overexpression, respectively. Immunoblots were quantified by densitometric analysis and normalized for β-actin. Numbers represent fold increase relative to the condition shown with bold number. All western blots are representative of three independent experiments. **g**, **h** Quantification of migration assays of MDA-MB-231 and MDA-MB-468 shGLO1 cells upon DUSP5 overexpression, respectively. Data were analyzed using one-way analysis of variance (ANOVA) followed by Dunnett post-hoc test or two-way ANOVA followed by Bonferroni post-hoc test and are shown as mean values ± SEM of three independent experiments. ns, not significant; *p < 0.05, **p < 0.01 and ***p < 0.001. **i** Key biological processes (green boxes) and regulatory pathways (yellow boxes) by which MG stress contributes to the metastatic phenotype. For the sake of clarity, the regulation of genes of the pro-metastatic signature exerted through the inhibition of MEK/ERK pathway (U0126 inhibitor) and SMAD1 (siSMAD1) is not represented (please see “Results” section for details)

Levels of ERK phosphorylation are dictated by the coordinated activity of protein kinases and phosphatases [27]. The (hyper)activation of MEK/ERK evidenced in breast cancer cells under MG stress led us to test the possibility that this persistent stimulation may be due to a defect in MAPK phosphatase expression in these cells. RNASeq data indeed showed decreased expression of dual-specificity (DUSPs) and phosphoprotein (PPP) phosphatase genes in GLO1-depleted cells (Additional file 2: Table S2). We quantified the mRNA levels of nine PPPs (either catalytic, structural or regulatory subunits) (Additional file 2: Figure S9A) and seven DUSPs (Fig. 7a and Additional file 2: Figure S9B) in MDA-MB-231 and MDA-MB-468 cells. DUSPs control the duration and magnitude of MAPK signaling activities. We selected DUSP1, 5, 8 and 16 for further validation because of their consistent down-regulation in MDA-MB-231 (Fig. 7a) and MDA-MB-468 cells (Additional file 2: Figure S9B) upon silencing with 2 GLO1 shRNAs. We validated the down-regulation of DUSP5 and 8 at the protein level in both cell lines (Fig. 7b). Following this, we focused on DUSP5 loss because of its known specificity for ERK1/2 and its significant decrease upon MG treatment at the mRNA and protein levels (Fig. 7c and d, respectively) in MDA-MB-231 and MDA-MB-468 cells. To gain insights into this new association between DUSP5 down-regulation and MG stress, we overexpressed DUSP5 in GLO1-depleted MDA-MB-231 and MDA-MB-468 cells. Under these conditions, we observed a significant P-ERK decrease associated with reduced SMAD1 and P-SMAD1 expression in MDA-MB-231 (Fig. 7e) and MDA-MB-468 cells (Fig. 7f). Upon DUSP5 overexpression, both GLO1-depleted cells showed a decreased migratory capacity (Fig. 7g and h and Additional file 2: Figure S10A and B) thus functionally linking DUSP5 regulation occurring under MG stress with pro-metastatic features in breast cancer cells. Everything included, we have then demonstrated that MG dicarbonyl stress endows breast cancer cells with genetic changes, notably affecting (i) ECM matrix reorganization, (ii) MEK/ERK cascade via cross-talk with SMAD1 transcriptional activity and (iii) DUSPs inhibition, which ultimately promoted their migratory and metastatic potential (Fig. 7i).

## Discussion

MG dicarbonyl stress has recently been associated with the aggressiveness of various malignancies including colon and breast carcinoma. However, the molecular mechanisms underlying these effects have not been clarified so far, particularly regarding MG-driven metastatic phenotype acquisition. Thanks to a comprehensive transcriptomic approach we identified a pro-metastatic signature associated with the MG stress condition, which notably comprised genes associated with ECM remodeling, and migration, two traits that are essential for metastasis.

Stromal cells are generally considered the main contributors to tumor ECM deposition and remodeling that favor tumor growth and invasion. In this study, we showed for the first time that cancer cells under MG stress directly contribute to major ECM changes. Differentially expressed genes in GLO1-depleted cells include several ECM components such as collagens, tenascin C and lumican. On the one hand, this finding corroborates recent studies implicating tumor cells in the expression of ECM components (i.e., collagens and fibronectin) that fulfill pro-tumoral and pro-metastatic functions at both primary and metastatic sites. On the other hand, an interesting parallel can be drawn with diabetes mellitus, where hyperglycemia results in the accumulation of MG and its protein adducts. In fact, GLO1-depleted myoblasts grown under a hyperglycemic condition display MG-induced elevated expression of collagens and increased fibrosis [28]. Elevated amounts of collagens secreted by MG-stressed tumor cells could be cross-linked by MG and thus generate specific mechanotransduction signals on tumor cells to favor their acquisition of a pro-invasive and pro-metastatic phenotype. In good accordance with this hypothesis, GLO1-depleted cancer cells show augmented ECM reorganization, anchorage-independent growth and increased migration/invasion ability; all inhibited in the presence of MG scavengers. Next to collagens, tenascin C is another remarkable cancer cell-derived ECM component overexpressed upon MG stress. Tenascin C induces epithelial-to-mesenchymal transition (EMT) changes in breast cancer cells [29] and promotes their survival and outgrowth at secondary organs, such as the lung [30]. An example consistent with the acquisition of an invasive phenotype upon MG stress is the up-regulation of CD24, a mucin-like adhesion molecule that enhances the metastatic potential of malignant cells and is shown to be a marker of poor prognosis in breast carcinomas [31]. It is noteworthy that MG-stress-driven fine-tuning of the ECM consisted of the up-regulation of pro-metastatic genes but also the down-regulation of specific components such as lumican the reduced expression of which has been associated with poor prognosis in patients with breast cancer [32]. Further studies will help us better understand the role of MG in pro-tumoral ECM remodeling through a direct effect on cancer cells but also on surrounding cells such as fibroblasts and endothelial cells.

Several pro-tumorigenic signaling pathways that are activated by ECM stiffness promote aerobic glycolysis [33]. Emerging evidence indicates that metabolic alterations and an abnormal ECM cooperatively drive cancer cell aggressiveness and treatment resistance [20, 33]. In this study, we connected MEK/ERK/SMAD1 signaling activation and ECM remodeling with the major and underestimated consequence of the glycolytic switch - MG stress. We propose that MG stress could be sustained through a positive regulatory loop induced by ECM quantitative and qualitative changes. In this study, MCF7-M cells that had undergone EMT transition and glycolytic switch had accumulation of MG adducts, GLO1 and NRF2 overexpression and a higher migratory capacity than parental cells, this latter being reversed using MG scavengers.

SMAD4 is a common transcriptional partner for activated SMADs, the loss of which predicts liver metastasis in colon cancer [34] and bad prognosis in patients with breast cancer [35]. We showed that upon dicarbonyl stress, breast cancer cells had a significant decrease in SMAD4 and had enhanced migratory capacity. Our previous data [12] and this study suggest that MG stress mimics TGFβ control in ECM remodeling and the EMT process. Consistent with the hypothesis of a similarity between the pro-tumor effects of TGFβ and MG stress is the loss of SMAD4, which has been shown to abolish TGFβ tumor-suppressive functions while maintaining its role as an EMT inducer [36]. Massagué’s group originally reported that BMP and ERK signaling inactivate SMAD1 through the phosphorylation of specific serine residues [25]. This mechanism has notably been invoked to explain how oncogenic RAS could override TGFβ tumor suppressive effects [37]. In other studies, RAS-ERK signals enhanced the transcriptional activity of SMAD1 in response to BMPs [38]. Mechanistically, we provided evidence that MG stress induces sustained activation of the MEK/ERK pathway that signals through SMAD1 independently of KRAS mutation status. Accordingly, either the inhibition of MEK or SMAD1 impacts on the expression of MG stress pro-metastatic signature and decreases the migratory potential of breast cancer cells. We further pointed out for the first time that MG significantly impacts on MAPK-mediated response to stress through the inhibition of DUSP catalytic activity in breast cancer cells.

The role of GLO1 in cancer progression remains controversial. In this study, GLO1 silencing favored a pro-migratory and metastatic phenotype, suggesting a tumor-suppressing role of GLO1. A recent study published by Guo and colleagues reported that inhibition of GLO1 promoted apoptosis and decreased invasion of MDA-MB-231 cells [39]. These seemingly contradictory results both point to the importance of determining the MG concentrations achieved when using the GLO1 silencing strategy. Indeed, we recently demonstrated that MG exerts an hormetic effect on cancer cells that is defined by low-dose stimulation and high-dose inhibition of tumor growth [9].

The significant anti-metastatic effect of carnosine designates MG stress as a promising target for therapeutic intervention in aggressive tumor cells that have undergone energy metabolic switch. To date, inhibitors of glycolysis have had only modest therapeutic efficacy in cancer. These unsatisfactory results may be related to the large heterogeneity reported for several glycolytic enzymes, as a consequence of different genes, splice forms and post- translational modifications. These latter make cancer cells react differently when challenged with anti-glycolytic agents [40]. Based on our results, we postulate that the use of MG scavengers to prevent the formation of protein and DNA adducts might be a more adaptive strategy to target highly glycolytic tumors. Importantly, carnosine reversed the activation of MEK/ERK MAPK signalization, thus further highlighting the therapeutic potential of MG scavengers across different malignancies. Despite the recent recognition of MG as an oncometabolite [41], contemplating the targeting of dicarbonyl stress as a promising strategy for cancer prevention and/or therapy is still in its early days and merits further studies.

## Conclusions

The main results of this study further emphasize the relevance of MG stress in the acquisition of a metastatic phenotype by breast cancer cells. Our data point to direct ECM remodeling by breast cancer cells and sustained activation of MAPK signaling as key molecular events underlying MG stress. This study improves our understanding of the connection between glycolytic switch and breast cancer aggressiveness and proposes MG scavenger molecules (such as carnosine and aminoguanidine) as potential novel treatment options in the management of metastatic breast cancer.

## Additional files

Additional file 1: Supplementary methods: RNA sequencing, shRNA transfection, siRNA transfection, Plasmids transfection, Immunofluorescence, RNA isolation and quantitative reverse transcription-PCR (qRT-PCR), Western blot, Flow cytometry analysis and Soft agar colony formation assays. (PDF 560 kb)

Additional file 2: **Figure S1**. RNA sequencing analysis of GLO1-depleted MDA-MB-231 cells. (A) MG extracellular concentrations were assessed over 48 h in conditioned medium of GLO1-depleted MDA-MB-231 cells using UPLC-MS/MS. (B) Volcano plots highlighting differentially expressed genes in shGLO1#1 and shGLO1#2 MDA-MB-231 cells. Orange and red dots represent genes differentially expressed significantly (*q* < 0.05 and log2 fold change (FC) > 1) for shGLO1 clones. Red dots represent genes of the pro-metastatic signature. (C) Tenascin C, Lumican and CD24 mRNA levels were assessed by qRT-PCR in MDA-MB-231 cells treated with MG 300 and 500 μM for 1 h. Data were analyzed using one-way ANOVA followed by Dunnett post-hoc test and shown as mean values ± SEM of three independent experiments. *p < 0.05, **p < 0.01 and ***p < 0.001. **Figure S2**. Dicarbonyl stress promotes anchorage-independent growth and invasion of breast cancer cells. Representative pictures of the colonies formed in a soft agar matrix by GLO1-silenced MDA-MB-231 cells treated with carnosine. **Figure S3**. Dicarbonyl stress promotes invasion of breast cancer cells. (A) Migration ability toward serum of GLO1-overexpressing shNT and shGLO1#1 MDA-MB-231 cells was assessed using Transwell filters. Representative filters are shown for each condition. (B) Quantification of migration assays of MDA-MB-231 shGLO1 cells upon GLO1 overexpression. (C) GLO1 protein level was assessed using immunoblot in GLO1-silenced MDA-MB-231 upon GLO1 overexpression. β-actin protein is used as loading control. Western blot is representative of three independent experiments. (D) Invasiveness ability of GLO1-depleted MDA-MB-231 toward serum was assessed using Transwell filters. Where indicated, cells were pre-treated with MG scavengers, carnosine and aminoguanidine, 24 h prior to the assay. Representative filters are shown for each condition. Scale bar represents 400 μm. (E) Quantification of invasiveness ability of GLO1-silenced MDA-MB-231 cells treated with carnosine and aminoguanidine. Data were analyzed using two-way ANOVA followed by Bonferroni post-hoc test and shown as mean values ± SEM of three independent experiments. ns, not significant; *p < 0.05, **p < 0.01 and ***p < 0.001. **Figure S4**. Highly migratory MCF7 cells (MCF7-M) display enhanced aerobic glycolysis. (A) Representative pictures of cultured MCF7 and MCF7-M cells. Scale bar represents 400 μm. (B) Extracellular acidification rates (ECAR) in MCF7 and MCF7-M cells using Seahorse flux analyzer. Glycolytic-related ECAR was calculated. Data were analyzed using Student’s *t* test and shown as mean values ± SEM of two independent experiments. **p < 0.01. **Figure S5**. Dicarbonyl stress promotes migration and anchorage-independent growth of MDA-MB-468 breast cancer cells. (A) GLO1 protein level in MDA-MB-468 shNT control and shGLO1#2 and #3 cells. β-actin protein is used as loading control. Western blot is representative of three independent experiments. (B) Lumican and CD24 mRNA levels were assessed by qRT-PCR in GLO1-depleted MDA-MB-468 cells. (C) Lumican and CD24 mRNA levels were assessed by qRT-PCR in MDA-MB-468 cells treated with MG 300 and 500 μM for 1 h. Data were analyzed using one-way ANOVA followed by Dunnett post-hoc test and shown as mean values ± SEM of three independent experiments. *p < 0.05, **p < 0.01 and ***p < 0.001. (D) Migration ability of GLO1-depleted MDA-MB-468 toward serum was assessed using Transwell filters. Where indicated, cells were pre-treated with carnosine 24 h prior to the assay. Representative filters are shown for each condition. Scale bar represents 400 μm. (E) Quantification of migratory ability of GLO1-silenced MDA-MB-468 cells treated with carnosine. Data were analyzed using two-way ANOVA followed by Bonferroni post-hoc test and shown as mean values ± SEM of three independent experiments. (F) Representative pictures of the colonies formed in a soft agar matrix by GLO1-silenced MDA-MB-468 cells treated with carnosine. (G) Quantification of colonies formed in a soft agar matrix by GLO1-silenced MDA-MB-468 cells treated with carnosine. Data were analyzed using two-way ANOVA followed by Bonferroni post-hoc test and shown as mean values ± SEM of three independent experiments. *p < 0.05, **p < 0.01 and ***p < 0.001. **Figure S6**. P-ERK localization in GLO1-depleted breast cancer cells. P-ERK immunofluorescence staining in GLO1-silenced MDA-MB-231 (A) and MDA-MB-468 (B) cells cultured in serum-free conditions. Data are representative of three independent experiments. Magnification × 630. Zoomed pictures (white square) are shown where indicated. **Figure S7**. GLO1 rescue impairs increased SMAD1 phosphorylation in GLO1-depleted MDA-MB-231 cells and SMAD1 activation favors the enhanced migration ability of MCF7-M cells. (A) (P-)SMAD1 (ser206) protein levels were assessed using immunoblot in shNT and shGLO1#1 MDA-MB-231 cells upon GLO1 overexpression. (B) P-SMAD1 (ser206) and SMAD1 protein level in MCF7 and MCF7-M cells cultured in serum-free conditions. (C) Migration ability toward serum of SMAD1-silenced MCF7 and MCF7-M cells was assessed using Transwell filters. Representative filters are shown for each condition. Scale bar represents 400 μm. (D) Quantification of migration assays of SMAD1-silenced MCF7 and MCF7-M cells. (E) SMAD1 protein level assessed by immunoblot to validate SMAD1-silencing using siRNAs in MCF7 and MCF7-M cells (related to panels B and C). β-actin or HSC70 were used as loading control. Immunoblots were quantified by densitometric analysis and normalized for β-actin. Numbers represent fold increase relative to the condition shown with bold number. All western blots are representative of three independent experiments. Data were analyzed using two-way ANOVA followed by Bonferroni post-hoc test and shown as mean values ± SEM of three independent experiments. *p < 0.05, **p < 0.01 and ***p < 0.001. **Figure S8**. SMAD1 activation promotes the pro-metastatic phenotype in MDA-MB-468 cells. A. SMAD1 target genes mRNA levels were assessed using qRT-PCR in SMAD1-silenced MDA-MB-468 shGLO1 cells. SMAD1 mRNA level was assessed to validate efficient SMAD1 siRNA silencing. (B) Migration ability toward serum of SMAD1-silenced MDA-MB-468 shGLO1 cells was assessed using Transwell filters. Representative filters are shown for each condition. Scale bar represents 400 μm. (C) Quantification of migration assays of SMAD1-silenced MDA-MB-468 shGLO1 cells. (D) SMAD1 protein level assessed by immunoblot to validate SMAD1-silencing using siRNAs in GLO1-depleted MDA-MB-468 cells (related to panels B and C). Western blots are representative of three independent experiments. Data were analyzed using two-way ANOVA followed by Bonferroni post-hoc test and shown as mean values ± SEM of three independent experiments. *p < 0.05, **p < 0.01 and ***p < 0.001. **Figure S9**. GLO1 depletion is associated with a global decrease of phosphatases expression in breast cancer cells. (A) mRNA levels of PPP2 phosphatases (from catalytic, structural and regulatory subunits) in GLO1-depleted MDA-MB-231 and MDA-MB-468 cells were assessed by qRT-PCR. (B) DUSP1, 5, 8, 10, 12, 14 and 16 phosphatases mRNA levels in GLO1-depleted MDA-MB-468 cells were quantified by qRT-PCR. Data were analyzed using one-way ANOVA followed by Dunnett post-hoc test and shown as mean values ± SEM of three independent experiments. ns, not significant; *p < 0.05, **p < 0.01 and ***p < 0.001. **Figure S10**. DUSP5 overexpression decreases the migratory capacity of GLO1-depleted breast cancer cells. Migration ability toward serum of DUSP5-overexpressing MDA-MB-231 (A) and MDA-MB-468 (B) cells was assessed using Transwell filters. Representative filters are shown for each condition. **Table S1**. Remarkable genes coding for ECM components and ECM regulators, the expression of which is significantly modulated in GLO1-depleted MDA-MB-231 breast cancer cells. Genes shown in bold have been validated at the protein and/or mRNA expression levels. ns, not significant. **Table S2**. DUSPs and PPPs subunits expression data from RNASeq in GLO1-depleted MDA-MB-231 cells. Fold change and false discovery rate (FDR) are shown for both shGLO1 clones. ns, not significant. **Table S3**. Primer sequences and probes used for quantitative reverse transcription-PCR (qRT-PCR). **Table S4**. Antibodies and dilution used for western blot experiments. (PDF 2523 kb)
