# Supplementary material for: Methylglyoxal, a glycolysis metabolite, triggers metastasis through MEK/ERK/SMAD1 pathway activation in breast cancer
Source: Breast Cancer Res. 2019 Jan 23;21:11. doi: 10.1186/s13058-018-1095-7 (PMC6343302; doi:10.1186/s13058-018-1095-7)
Supplement: Supplementary file 1 — Supplementary methods: RNA sequencing, shRNA transfection, siRNA transfection, Plasmids transfection, Immunofluorescence, RNA isolation and quantitative reverse transcription-PCR (qRT-PCR), Western blot, Flow cytometry analysis and Soft agar colony formation assays. (PDF 560 kb) [file 13058_2018_1095_MOESM1_ESM.pdf]

SUPPLEMENTARY METHODS

RNA sequencing. RNA sequencing was performed on libraries prepared from total RNA samples. Three biological replicates were analyzed for each condition (shNT, shGLO1#1 and shGLO1#2). RNA integrity was verified on a Bioanalyser 2100 with RNA 6000 Nano chips (Agilent). RNA integrity number score was above 8 for every sample. Libraries were prepared using Truseq® stranded mRNA Sample Preparation Kits (Illumina) following manufacturer's instructions. PolyA RNAs were isolated from 1 µg of total RNA with polyT-coated magnetic beads, then chemically fragmented and used as template for cDNA synthesis using Superscript II reverse transcriptase (Invitrogen) and random hexamers as primers. Strand specificity was achieved by substituting dUTP for dTTP during second strand synthesis. After A-tailing and ligation to indexed adapters, fragments with adapters were purified and enriched by PCR to generate the final cDNA libraries. Libraries were multiplexed and sequenced on an Illumina NextSeq500 sequencer to generate ~20,000,000 paired-end reads of 76 bases per library. Raw reads demultiplexed and adapter-trimmed using Illumina bcl2fastq conversion software v2.17 were aligned to the human hg19 reference genome using Tophat v2.1.1(1). Reads were first aligned to a virtual transcriptome generated based on hg19 gene models, then reads that did not fully map were aligned to the genome, spliced as needed. Quality of the sequencing data was successfully controlled using FastQC (http://www.bioinformatics.babraham.ac.uk/projects/fastqc/) and Picard tools (http://broadinstitute.github.io/picard/). Raw gene counts were generated using HTseq count (2) in "union" mode and differential expression analysis was performed using the DESeq2 R package (3) comparing either shGLO1#1 or shGLO1#2 to shNT replicates. The average number of reads approached or exceeded 20 millions in all

samples. Alignment of transcripts to the genome indicated that 11653, 11910 and 11813 genes were expressed in shNT and shGLO1#1 and shGLO1#2, respectively.For differential gene expression, absolute fold change >2 and q-value < 0.05 filters were applied. The expression of 2 genes commonly used as calibrators was not significantly affected under shGLO1 condition when compared with shNT cells (GAPDH: fold changes 0.8 and 1.1 and ACTB: 0.8 and 0.9 for shGLO1#1 and #2, respectively). As expected, GLO1 level was significantly decreased in shGLO1#1 and #2 cells relatively to control cells.Lists of genes differentially expressed were imported in the ToppGene Suite for analysis, independently for both shGLO1 clones(4).The data discussed in this publication have been deposited in NCBI's Gene Expression Omnibus and are accessible through GEO Series accession number GSE122229 (https://www.ncbi.nlm.nih.gov/geo/query/acc.cgi?acc=GSE122229).

shRNA transfection. Lentiviral vectors were generated with the help of the GIGA Viral Vectors platform (University of Liège) as previously described(5). All shRNAs plasmids were from Sigma: TRCN0000118627 (#1), TRCN0000118631 (#2), TRCN0000118630 (#3), and non-target (NT, anti-eGFP) (SHC005). Cells were stably transduced and selected with puromycin (0.5µg/ml, Sigma).

siRNA transfection.SMAD1 (5'-GGAACUGCAACUACCAUCAUGGAUU-3') small interfering RNA (siRNA) and siGl3 irrelevant used as control were synthesized by Eurogentec. Calcium phosphate-mediated transfections were performed using 20nM of each siRNA.

Plasmids transfection. pRP[Exp]-Puro-EF1A>hDUSP5 (pDUSP5) andpLV[Exp]-Bsd-EF1A>hGLO1 (pGLO1) and their respective control vector,empty vector (pEV) and GFP expression plasmid (pGFP),were synthetized by VectorBuilder (Santa

Clara, CA, USA). Cell transfection was performed using Lipofectamine (ThermoFisher Scientific) according to manufacturer's instructions.

Immunofluorescence. Cells were plated on coverslips in 24-well plates and fixed/permeabilized with cold methanol/acetone (for SMAD1 and P-ERK). After blocking (3% BSA for 30min), slides were incubated with rabbit anti-SMAD1 (Cell Signaling, 1:100) and anti-P-ERK (Cell Signaling, 1:100) antibodies diluted in 1% BSA overnight at 4°C. After washing with PBS, slides were incubated with anti-rabbit IgG AlexaFluor488 conjugated secondary antibodies (Life Technologies, 1:1000) for 1h at RT. Nuclei were stained using DAPI (EMD Chemicals). Coverslips were mounted on glass slides and observed using confocal microscope (Leica SP5).

RNA isolation and quantitative reverse transcription-PCR (qRT-PCR). RNA extraction, reverse transcription and Q-PCR were performed as previously described (5). cDNA were mixed with primers, probe (Universal ProbeLibrary System, Roche) and 2x FastStart Universal Probe Master Mix (Roche). Q-PCR were performed using the 7300 Real Time PCR System and the corresponding manufacturer's software (Applied Biosystems). Relative gene expression was normalized to 18S rRNA. Primers sequences are presentedin Supplementary Table S2 (Eurogentec). Three technical replicates of each sample have been performed and data are presented as mean ± SEM of minimum 3 biological replicates.

Western Blot. Briefly, cells were extracted in SDS 1% buffer containing protease and phosphatase inhibitors (Roche). Cell-free deposited proteins were obtained after total removal of cell with Versene solution and lysis of extracellular matrix proteins. Protein concentrations were determined using the bicinchoninic acid assay (Pierce). Twenty µg of proteins were separated by SDS-PAGE and transferred to PVDF membranes. Then, membranes were incubated with primary antibodies overnight at

4°C and to appropriate secondary antibody at RT for 1 hour. Antibodies are listed in Supplementary Table S3. The immunoreactive bands were visualized using ECL Western Blotting substrate (Pierce). Immunoblots were quantified by densitometric analysis and normalized for β-actin using ImageJ software. A representative western blot of three independent biological replicates is shown.

Flow cytometry analysis. Cells were trypsinized and incubated with anti-CD24 antibody (1/100, BD Biosciences, San Jose, CA, USA) or with the control IgG in the dark. After 30 minutes, cells were analyzed by flow cytometry (BD Biosciences FACSCanto). Median fluorescence intensity (MFI) was calculated according to the following formula: $MFI = \frac{median\ \ CD24 - median\ \ IgG}{median\ \ IgG}$. All data are presented as the mean ± SEM of four biological replicates.

Soft agar colony formation assays. Soft agar colony formation assays were performed as described previously (6). Briefly, 2500 MDA-MB-231 cells or 1000 MDA-MB-468 cells were plated in a 0.4% agar mixture. Two times per week, fresh medium containing carnosine was added on agar layer. After 21 days, colonies were stained with crystal violet and further quantified using ImageJ software. Three biological replicates with four wells per condition were analyzed.

REFERENCES TO SUPPLEMENTARY MATERIAL AND METHODS SECTION

1. Kim D, Pertea G, Trapnell C, Pimentel H, Kelley R, Salzberg SL. TopHat2: accurate alignment of transcriptomes in the presence of insertions, deletions and gene fusions. Genome biology **2013**;14:R36
2. Anders S, Pyl PT, Huber W. HTSeq--a Python framework to work with high-throughput sequencing data. Bioinformatics **2015**;31:166-9
3. Love MI, Huber W, Anders S. Moderated estimation of fold change and dispersion for RNA-seq data with DESeq2. Genome biology **2014**;15:550
4. Chen J, Bardes EE, Aronow BJ, Jegga AG. ToppGene Suite for gene list enrichment analysis and candidate gene prioritization. Nucleic acids research **2009**;37:W305-11

5. Nokin MJ, Durieux F, Peixoto P, Chiavarina B, Peulen O, Blomme A*, et al.* Methylglyoxal, a glycolysis side-product, induces Hsp90 glycation and YAP-mediated tumor growth and metastasis. eLife **2016**;5
6. Borowicz S, Van Scoyk M, Avasarala S, Karuppusamy Rathinam MK, Tauler J, Bikkavilli RK*, et al.* The soft agar colony formation assay. Journal of visualized experiments : JoVE **2014**:e51998
