# Supplementary material for: Methylglyoxal, a glycolysis metabolite, triggers metastasis through MEK/ERK/SMAD1 pathway activation in breast cancer
Source: Breast Cancer Res. 2019 Jan 23;21:11. doi: 10.1186/s13058-018-1095-7 (PMC6343302; doi:10.1186/s13058-018-1095-7)
Supplement: Supplementary file 2 — Figure S1. RNA sequencing analysis of GLO1-depleted MDA-MB-231 cells. (A) MG extracellular concentrations were assessed over 48 h in conditioned medium of GLO1-depleted MDA-MB-231 cells using UPLC-MS/MS. (B) Volcano plots highlighting differentially expressed genes in shGLO1#1 and shGLO1#2 MDA-MB-231 cells. Orange and red dots represent genes differentially expressed significantly (q < 0.05 and log2 fold change (FC) > 1) for shGLO1 clones. Red dots represent genes of the pro-metastatic signature. (C) Tenascin C, Lumican and CD24 mRNA levels were assessed by qRT-PCR in MDA-MB-231 cells treated with MG 300 and 500 μM for 1 h. Data were analyzed using one-way ANOVA followed by Dunnett post-hoc test and shown as mean values ± SEM of three independent experiments. *p < 0.05, **p < 0.01 and ***p < 0.001. Figure S2. Dicarbonyl stress promotes anchorage-independent growth and invasion of breast cancer cells. Representative pictures of the colonies formed in a soft agar matrix by GLO1-silenced MDA-MB-231 cells treated with carnosine. Figure S3. Dicarbonyl stress promotes invasion of breast cancer cells. (A) Migration ability toward serum of GLO1-overexpressing shNT and shGLO1#1 MDA-MB-231 cells was assessed using Transwell filters. Representative filters are shown for each condition. (B) Quantification of migration assays of MDA-MB-231 shGLO1 cells upon GLO1 overexpression. (C) GLO1 protein level was assessed using immunoblot in GLO1-silenced MDA-MB-231 upon GLO1 overexpression. β-actin protein is used as loading control. Western blot is representative of three independent experiments. (D) Invasiveness ability of GLO1-depleted MDA-MB-231 toward serum was assessed using Transwell filters. Where indicated, cells were pre-treated with MG scavengers, carnosine and aminoguanidine, 24 h prior to the assay. Representative filters are shown for each condition. Scale bar represents 400 μm. (E) Quantification of invasiveness ability of GLO1-silenced MDA-MB-231 cells treated with carno [file 13058_2018_1095_MOESM2_ESM.pdf]

Supplementary Figure S1

A.

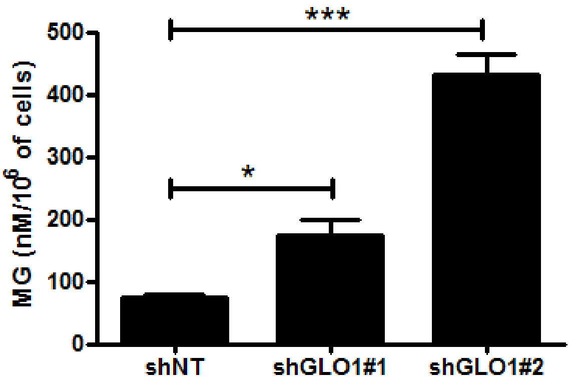

B.

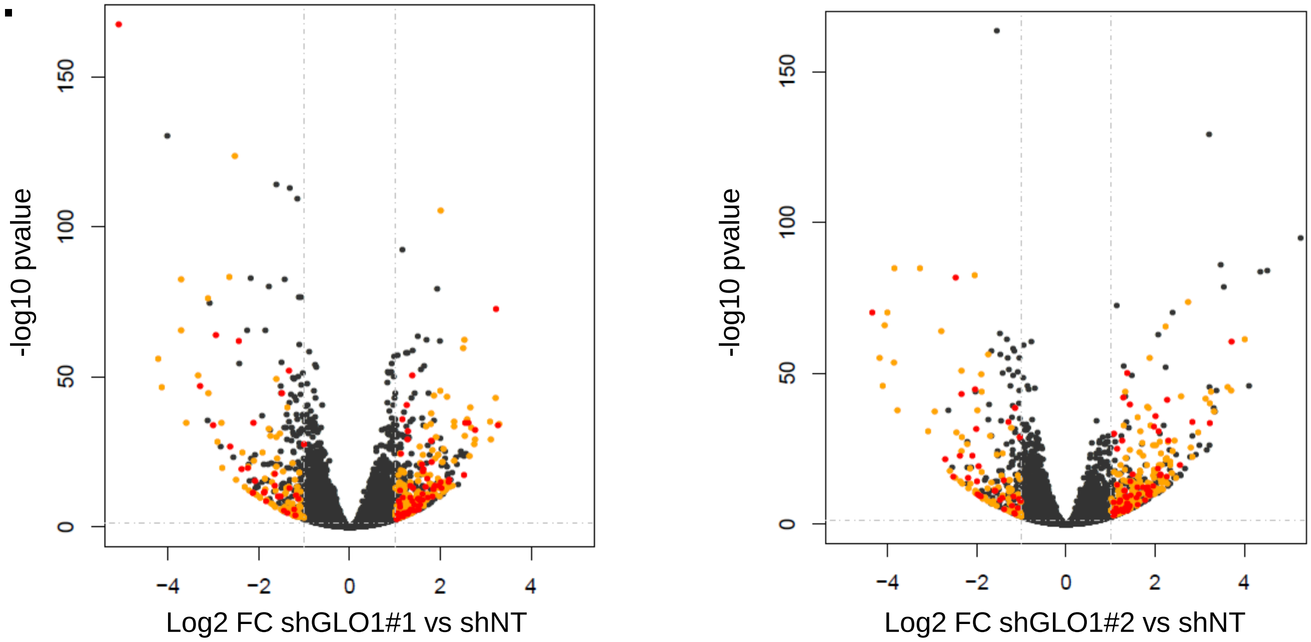

C.

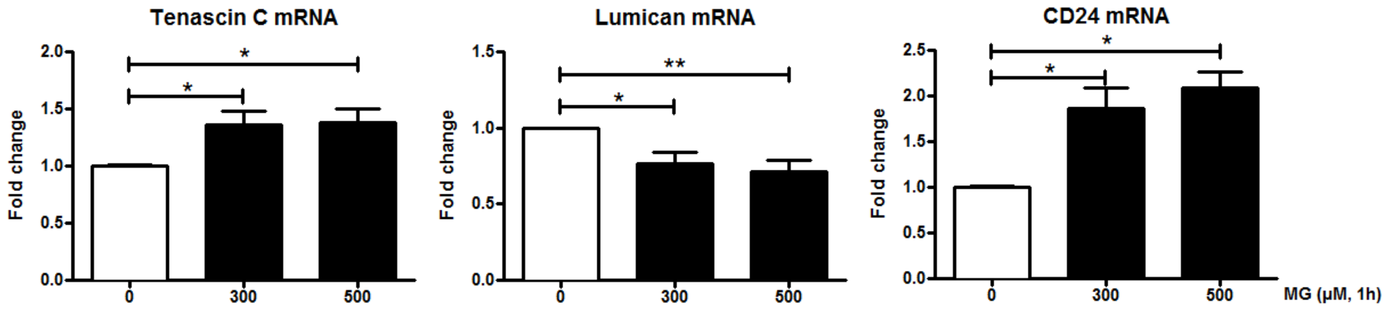

Supplementary Figure S1.RNA sequencing analysis of GLO1-depleted MDA-MB-231 cells. A. MG extracellular concentrations were assessed in forty-eight hours conditioned medium of GLO1-depleted MDA-MB-231 cells using UPLC-MS/MS.**B.** Volcano plots highlighting differentially expressed genes in shGLO1#1 and shGLO1#2 MDA-MB-231 cells. Orange and red dots represent genes differentially expressed significantly ($q < 0.05$ and log2 fold change (FC) >1) for shGLO1 clones. Red dots represent genes of the pro-metastatic signature.**C.**Tenascin C, Lumican and CD24 mRNA levels were assessed by qRT-PCR in MDA-MB-231 cells treated with MG 300 and 500µM during 1h. Data were analyzed using one-way ANOVA followed by Dunnet post-test and shown as mean values ± SEM of three independent experiments.*$p<0.05$, **$p<0.01$ and ***$p<0.001$.

Supplementary Figure S2

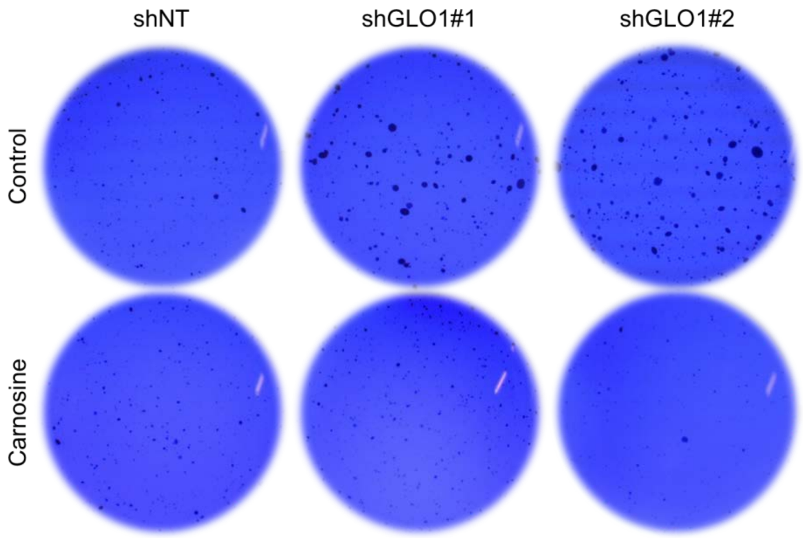

Supplementary Figure S2. Dicarbonyl stress promotes anchorage independent growth and invasion of breast cancer cells. Representative pictures of the colonies formed in a soft agar matrix by GLO1-silenced MDA-MB-231 cells treated with carnosine.

Supplementary Figure S3

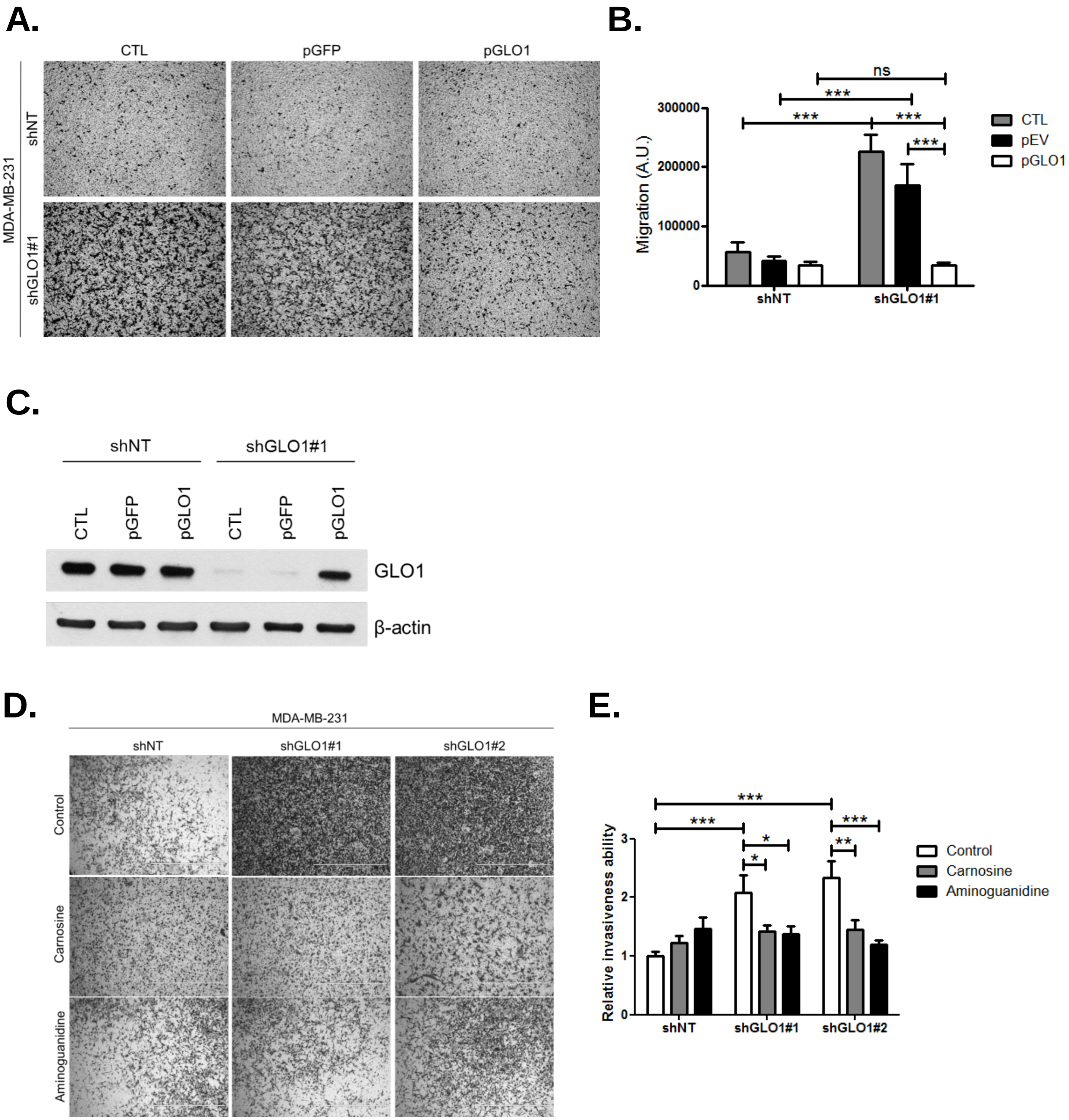

Supplementary Figure S3. Dicarbonyl stress promotes invasion of breast cancer cells. A. Migration ability toward serum of GLO1-overexpressing shNT and shGLO1#1 MDA-MB-231 cells was assessed using transwell filters. Representative filters are shown for each condition. **B.** Quantification of migration assays of MDA-MB-231 shGLO1 cells upon GLO1 overexpression. **C.** GLO1 protein level was assessed using immunoblot in GLO1-silenced MDA-MB-231 upon GLO1 overexpression. β-actin protein is used as loading control. Western blot is representative of 3 independent experiments. **D.**Invasiveness ability of GLO1-depleted MDA-MB-231 toward serum was assessed using transwell filters. Where indicated, cells were pre-treated with MG scavengers, carnosine and aminoguanidine, 24 hr prior the assay. Representative filters are shown for each condition. Scale bar represents 400 µm. **E.** Quantification of invasiveness ability of GLO1-silenced MDA-MB-231 cells treated with carnosine and aminoguanidine. Data were analyzed using two-way ANOVA followed by Bonferroni post-test and shown as mean values ± SEM of three independent experiments. ns = not significant, *p<0.05, **p<0.01 and ***p<0.001.

Supplementary Figure S4

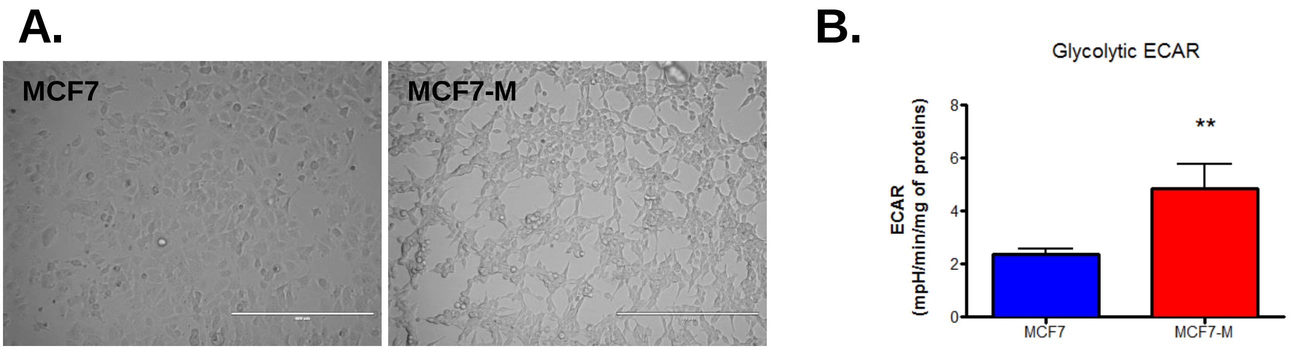

Supplementary Figure S4. Highly migratory MCF7 cells (MCF7-M) display enhanced aerobic glycolysis. A. Representative pictures of cultured MCF7 and MCF7-M cells. Scale bar represents 400 µm. **B.** Extracellular acidification rates (ECAR) in MCF7 and MCF7-M cells using Seahorse flux analyzer. Glycolytic related-ECAR was calculated. Data were analyzed using student's t test and shown as mean values ± SEM of two independent experiments. $^{**}p<0.01$.

Supplementary Figure S5

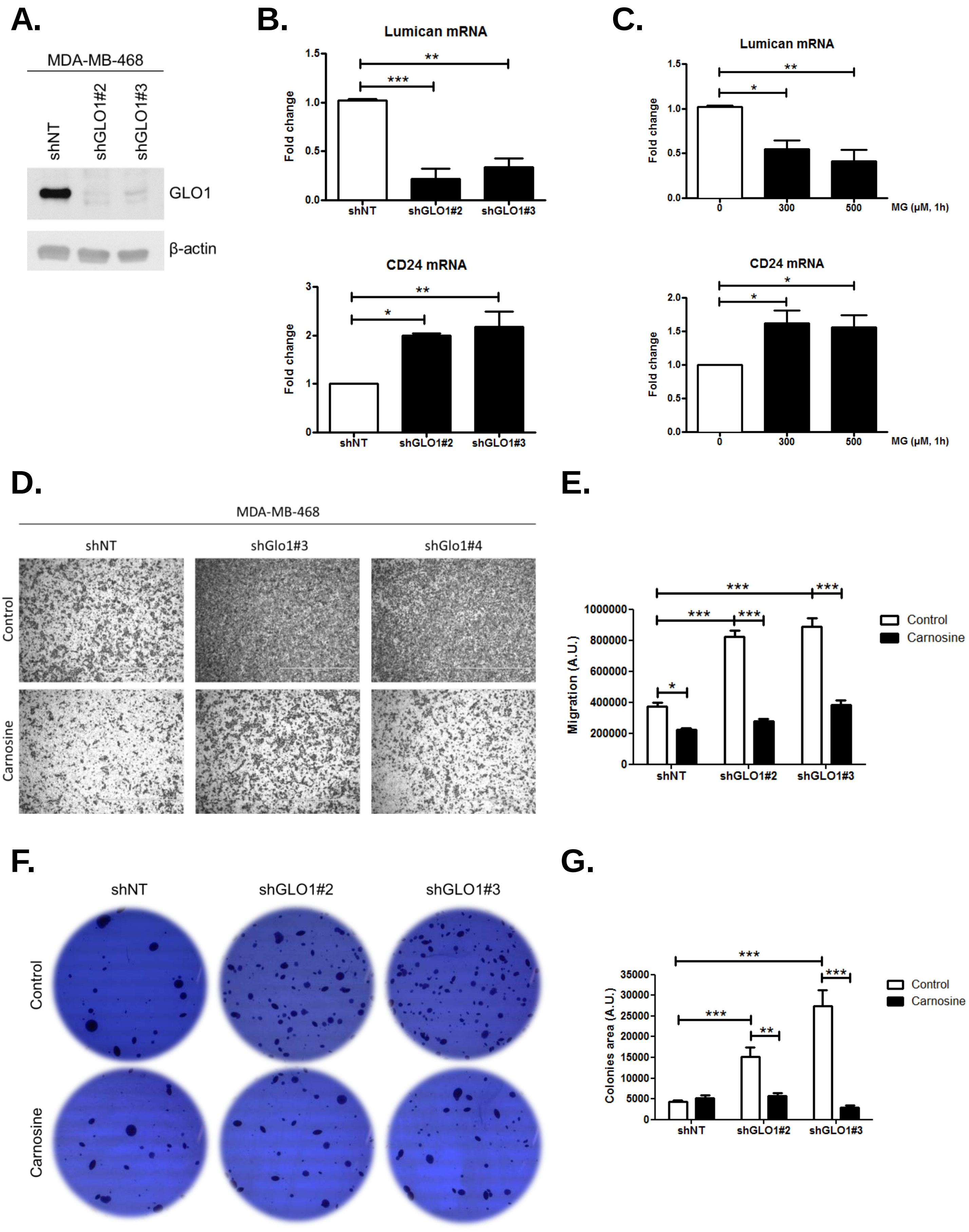

Supplementary Figure S5. Dicarbonyl stress promotes migration and anchorage independent growth of MDA-MB-468 breast cancer cells. A. GLO1protein level in MDA-MB-468 shNT control and shGLO1#2 and #3 cells. β-actin protein is used as loading control. Western blot is representative of 3 independent experiments. **B.**Lumican and CD24 mRNA levels were assessed by qRT-PCR in GLO1-depleted MDA-MB-468 cells. **C.**Lumican and CD24 mRNA levels were assessed by qRT-PCR in MDA-MB-468 cells treated with MG 300 and 500µM during 1h. Data were analyzed using one-way ANOVA followed by Dunnet post-test and shown as mean values ± SEM of three independent experiments.*p<0.05, **p<0.01 and ***p<0.001. **D.** Migration ability of GLO1-depleted MDA-MB-468 toward serum was assessed using transwell filters. Where indicated, cells were pre-treated with carnosine 24 hr prior the assay. Representative filters are shown for each condition. Scale bar represents 400 µm. **E.** Quantification of migratory ability of GLO1-silenced MDA-MB-468 cells treated with carnosine. Data were analyzed using two-way ANOVA followed by Bonferroni post-test and shown as mean values ± SEM of three independent experiments. **F.** Representative pictures of the colonies formed in a soft agar matrix by GLO1-silenced MDA-MB-468 cells treated with carnosine. **G.** Quantification of colonies formed in a soft agar matrix by GLO1-silenced MDA-MB-468 cells treated with carnosine. Data were analyzed using two-way ANOVA followed by Bonferroni post-test and shown as mean values ± SEM of three independent experiments. *p<0.05, **p<0.01 and ***p<0.001.

Supplementary Figure S6

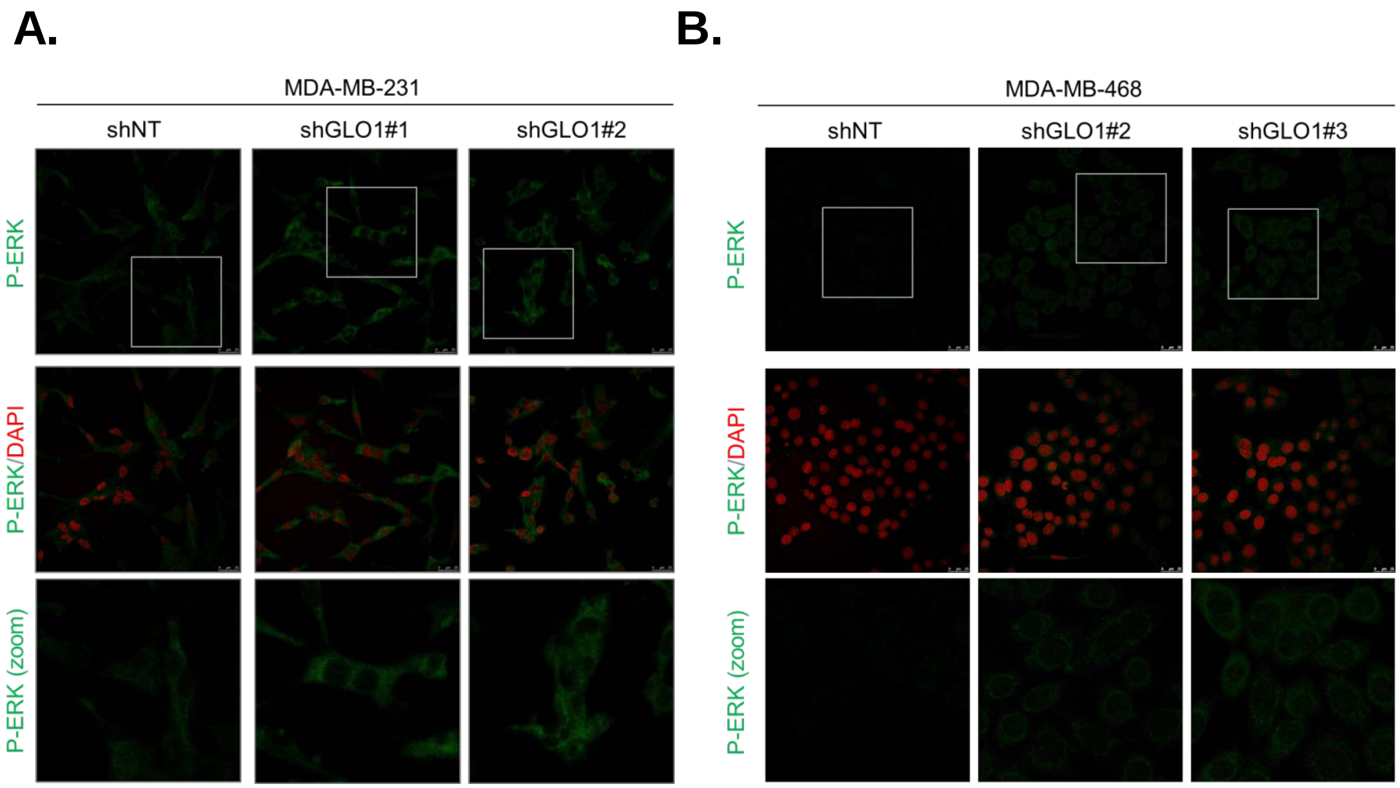

Supplementary Figure S6. P-ERK localization in GLO1-depleted breast cancer cells. P-ERK immunofluorescence staining in GLO1-silenced MDA-MB-231 (**A**.) and MDA-MB-468 (**B.**)cells cultured in serum free condition. Data are representative of three independent experiments. Magnification 630x. Zoomed pictures (white square) are shown where indicated.

Supplementary Figure S7

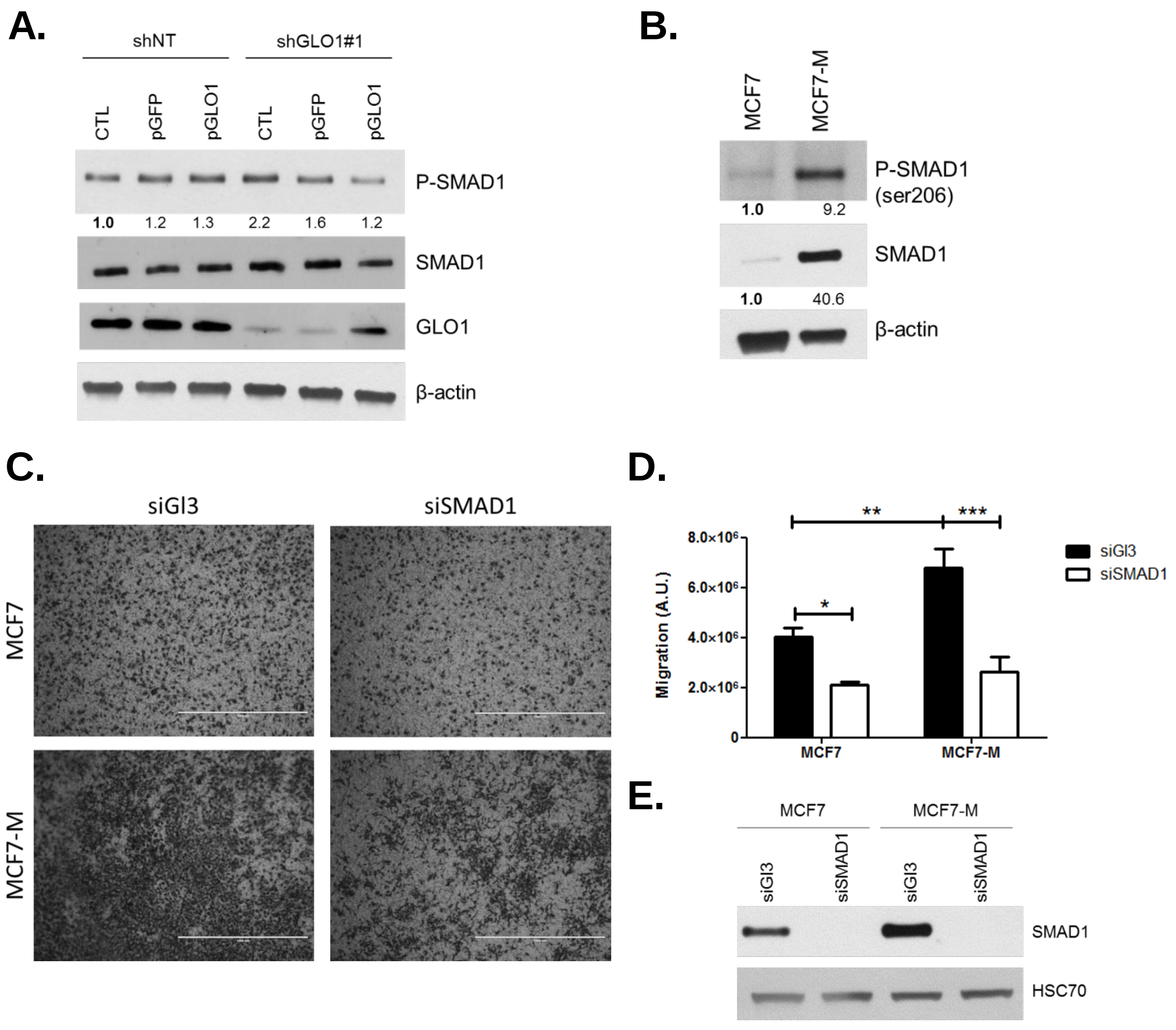

Supplementary Figure S7. GLO1 rescue impairs increased SMAD1 phosphorylation in GLO1-depleted MDA-MB-231 cells and SMAD1 activation favors the enhanced migration ability of MCF7-M cells. A. (P-)SMAD1 (ser206) protein levels were assessed using immunoblot in shNT and shGLO1#1 MDA-MB-231 cells upon GLO1 overexpression. **B.** P-SMAD1 (ser206) and SMAD1 protein level in MCF7 and MCF7-M cells cultured in serum free condition. **C.** Migration ability toward serum of SMAD1-silenced MCF7 and MCF7-M cells was assessed using transwell filters. Representative filters are shown for each condition. Scale bar represents 400 µm. **D.** Quantification of migration assays of SMAD1-silenced MCF7 and MCF7-M cells. **E.** SMAD1 protein level assessed by immunoblot to validate SMAD1-silencing using siRNAs in MCF7 and MCF7-M cells (related to B. and C. panels). β-actin or HSC70 were used as loading control. Immunoblots were quantified by densitometric analysis and normalized for β-actin. Numbers represent fold increase relative to the condition shown with bold number. All western blotsare representative of 3 independent experiments. Data were analyzed using two-way ANOVA followed by Bonferroni post-test and shown as mean values ± SEM of three independent experiments. *$p<0.05$, **$p<0.01$ and ***$p<0.001$.

Supplementary Figure S8

A.

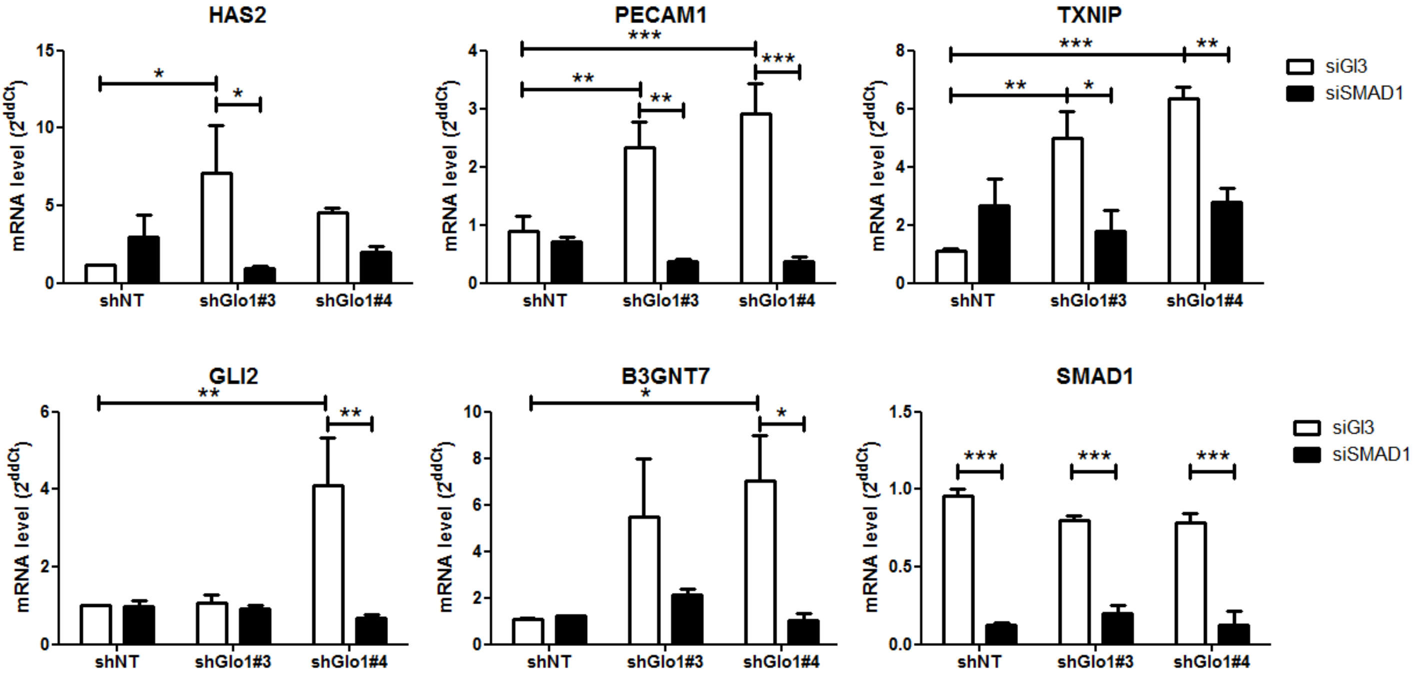

B.

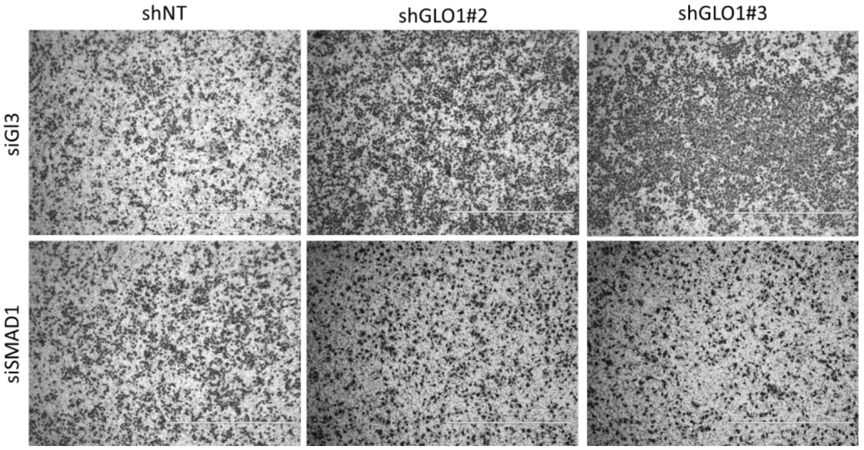

C.

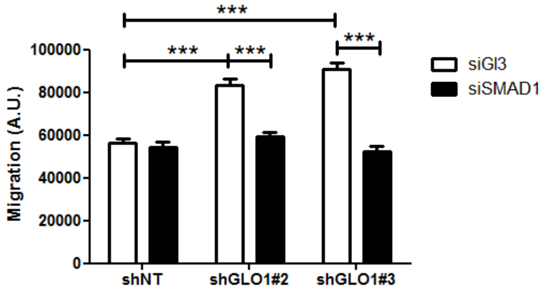

D.

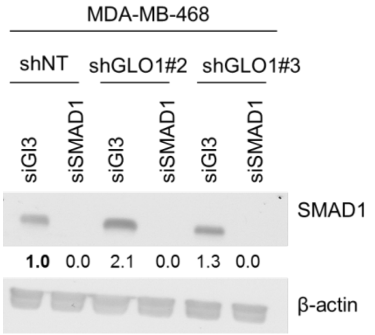

Supplementary Figure S8. SMAD1 activation promotes the pro-metastatic phenotype in MDA-MB-468 cells. A. SMAD1 target genes mRNA levels were assessed using qRT-PCR in SMAD1-silenced MDA-MB-468 shGLO1 cells. SMAD1 mRNA level was assessed to validate efficient SMAD1 siRNA silencing. **B.** Migration ability toward serum of SMAD1-silenced MDA-MB-468 shGLO1 cells was assessed using transwell filters. Representative filters are shown for each condition. Scale bar represents 400 µm. **C.** Quantification of migration assays of SMAD1-silenced MDA-MB-468 shGLO1 cells. **D.** SMAD1 protein level assessed by immunoblot to validate SMAD1-silencing using siRNAs in GLO1-depleted MDA-MB-468 cells (related to B. and C. panels). Western blots are representative of 3 independent experiments. Data were analyzed using two-way ANOVA followed by Bonferroni post-test and shown as mean values ± SEM of three independent experiments. *$p<0.05$, **$p<0.01$ and ***$p<0.001$.

Supplementary Figure S9

A.

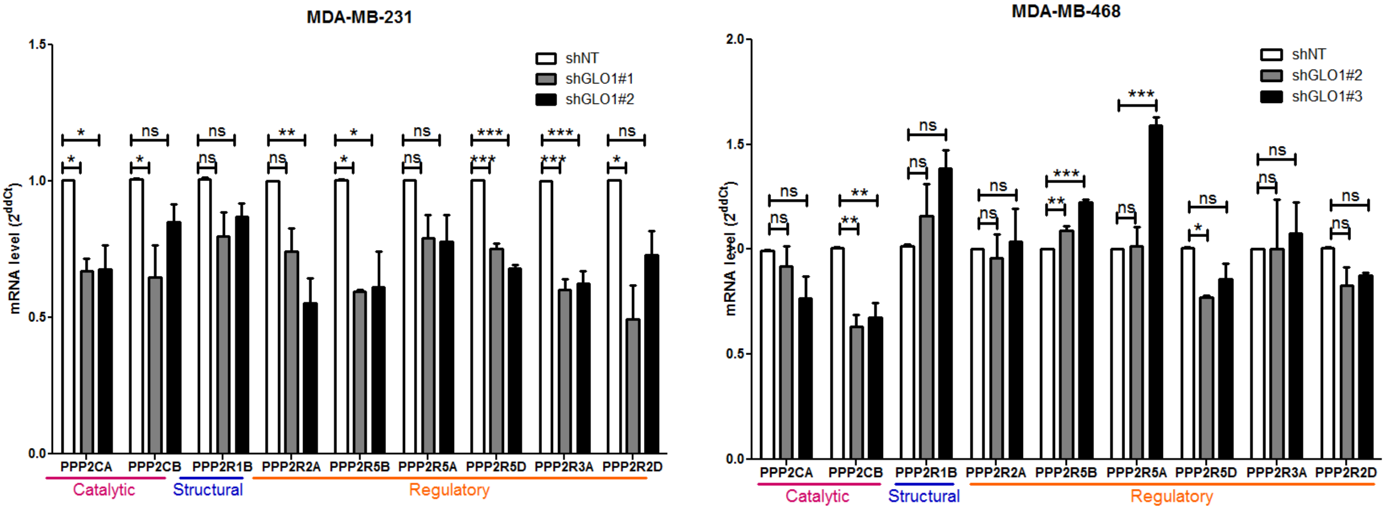

B.

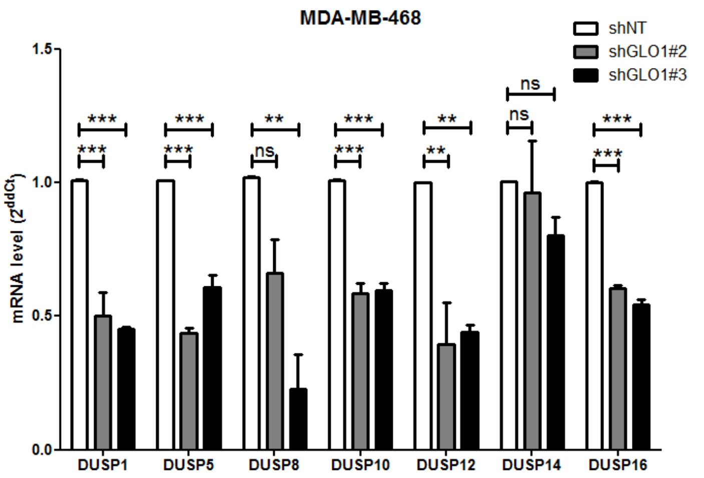

Supplementary Figure S9. GLO1 depletion is associated with a global decrease of phosphatases expression in breast cancer cells. A. mRNA levels ofPPP2 phosphatases (from catalytic, structural and regulatory subunits) in GLO1-depleted MDA-MB-231 and MDA-MB-468 cells were assessed by qRT-PCR. **B.** DUSP1, 5, 8, 10, 12, 14 and 16 phosphatases mRNA levels in GLO1-depleted MDA-MB-468 cells were quantified by qRT-PCR. Data were analyzed using one-way ANOVA followed by Dunnet post-test and shown as mean values ± SEM of three independent experiments.ns = not significant, *p<0.05, **p<0.01 and ***p<0.001.

Supplementary Figure S10

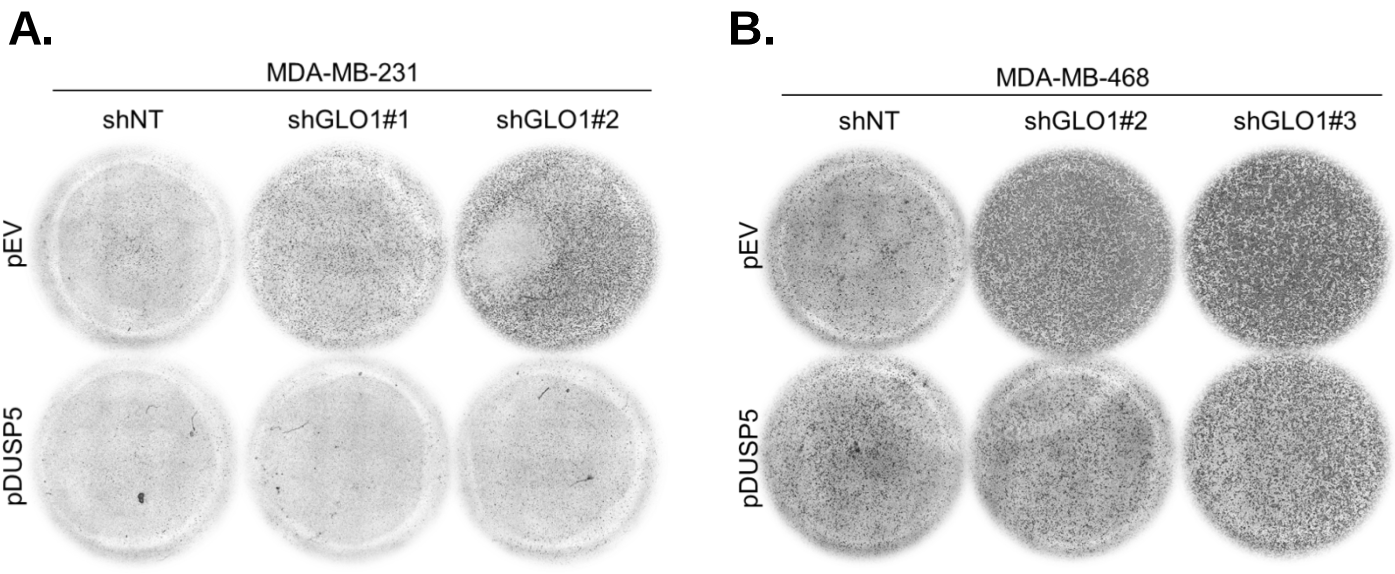

Supplementary Figure S10. DUSP5 overexpression decreases the migratory capacity of GLO1-depleted breast cancer cells. Migration ability toward serum of DUSP5-overexpressing MDA-MB-231 (**A**) and MDA-MB-468 (**B**) cells was assessed using transwell filters. Representative filters are shown for each condition.

Supplementary Table S1. Remarkable genes coding for ECM components and ECM regulators which expression is significantly modulated in GLO1-depleted MDA-MB-231 breast cancer cells. Genes shown in bold have been validated at the protein and/or mRNA expression levels. ns = not significant.

Genes	Gene name	**shGLO1#1** fold change, (fdr<0.001)	**shGLO1#2** fold change, (fdr<0.001)
ECM components			
• *Collagens*			
**Collagen type IV, alpha 3 chain**	**COL4A3**	**- 8.00**	**- 5.21**
Collagen type IV, alpha 4 chain	COL4A4	- 7.67	- 5.10
**Collagen type VI, alpha 1 chain**	**COL6A1**	**+ 4.00**	**+ 4.35**
Collagen type VI, alpha 2 chain	COL6A2	+ 4.53	+ 9.32
**Collagen type VI, alpha 3 chain**	**COL6A3**	**+ 9.32**	**+ 2.09**
Collagen type V, alpha 1 chain	COL5A1	+ 2.17	+ 2.20
Collagen type VII, alpha 1 chain	COL7A1	+ 2.41	+ 2.38
• *Proteoglycans :*			
Syndecan 1	SDC1	+ 2.01	ns
Syndecan 2	SDC2	ns	+ 2.66
**Tenascin C**	**TNC**	**+ 2.41**	**+ 2.43**
**Lumican**	**LUM**	**- 3.68**	**- 6.50**
• *Other ECM proteins:*			
Fibronectin 1	FN1	+ 2.57	+ 1.45
Laminin subunit alpha 4	LAMA4	+ 2.43	+ 2.69
**Transforming growth factor-beta-induced protein**	**TGFBI**	**+ 2.58**	**+ 2.10**
Matrilin 2	MATN2	- 4.53	- 1.66
Pleiotrophin	PTN	+ 6.11	+ 3.01
ECM receptors			
• *Integrins :*			
Integrin beta 2	ITGB2	- 3.14	- 2.27
Integrin beta 8	ITGB8	+ 3.03	+ 4.14
• *Non-integrins :*			
Ephrin B1	EPHB1	- 2.58	- 2.62
Ephrin B3	EPHB3	+ 2.45	+ 3.68
Plexin A4	PLXNA4	+ 3.92	+ 3.48
C-X-C chemokine receptor type 4	CXCR4	- 33.82	- 5.54
**CD24 molecule**	**CD24**	**+ 5.82**	**+ 4.89**
Cell-cell adhesion			
Melanoma cell adhesion molecule	MCAM	- 2.22	- 1.69
Intercellular adhesion molecule 1	ICAM1	- 2.00	- 2.04
**Platelet endothelial cell adhesion molecule**	**PECAM1**	**+ 2.16**	**+ 2.48**
E cadherin	CDH1	- 9.78	- 20.25
N cadherin	CDH2	- 3.68	- 4.59
Adhesion G protein-coupled receptor G1	ADGRG1	+ 2.23	+ 2.57
Adhesion G protein-coupled receptor G2	ADGRG2	+ 2.62	+ 2.22
MMPs and other modulators of ECM			
Matrix metallopeptidase 15	MMP15	-2.58	ns
Matrix metallopeptidase 24	MMP24	- 2.33	- 2.79
ADAM metallopeptidase domain 12	ADAM12	ns	+ 2.03
Hyaluronan synthase 2	HAS2	+ 2.51	+ 4.79
Rho GTPase J	RHOJ	+ 5.98	+ 4.82

Supplementary Table S2. DUSPs and PPPs subunits expression data from RNASeq in GLO1-depleted MDA-MB-231 cells. Fold change and false discovery rate (FDR) are shown for both shGLO1 clones. ns = not significant.

<table>
<tr><th rowspan="2">Gene</th><th rowspan="2">Name</th><th colspan="2">shGlo1#1</th><th colspan="2">shGlo1#2</th></tr>
<tr><th>Fold change</th><th>FDR</th><th>Fold change</th><th>FDR</th></tr>
<tr><td>DUSP1</td><td>Dual specificity protein phosphatase 1</td><td>-2,83</td><td>< 0.001</td><td colspan="2">ns</td></tr>
<tr><td>DUSP5</td><td>Dual specificity protein phosphatase 5</td><td>-1,87</td><td>< 0.001</td><td>-1,45</td><td>< 0.001</td></tr>
<tr><td>DUSP8</td><td>Dual specificity protein phosphatase 8</td><td>-2,64</td><td>< 0.001</td><td>-2,14</td><td>< 0.001</td></tr>
<tr><td>DUSP10</td><td>Dual specificity protein phosphatase 10</td><td>-2,64</td><td>< 0.001</td><td>-1,53</td><td>< 0.001</td></tr>
<tr><td>DUSP12</td><td>Dual specificity protein phosphatase 12</td><td>-1,3</td><td>< 0.001</td><td colspan="2">ns</td></tr>
<tr><td>DUSP14</td><td>Dual specificity protein phosphatase 14</td><td>-1,68</td><td>< 0.001</td><td colspan="2">ns</td></tr>
<tr><td>DUSP16</td><td>Dual specificity protein phosphatase 16</td><td>-1,25</td><td>0.046</td><td>-1,55</td><td>< 0.001</td></tr>
<tr><td>PPP2CA</td><td>Serine/threonine-protein phosphatase 2A catalytic subunit alpha isoform</td><td>-1,19</td><td>< 0.001</td><td colspan="2">ns</td></tr>
<tr><td>PPP2R1B</td><td>Protein phosphatase 2 scaffold subunit A beta</td><td>-1,17</td><td>0.034</td><td colspan="2">ns</td></tr>
<tr><td>PPP2R2A</td><td>Serine/threonine-protein phosphatase 2A regulatory subunit B alpha isoform</td><td colspan="2">ns</td><td colspan="2">ns</td></tr>
<tr><td>PPP2R5B</td><td>Serine/threonine-protein phosphatase 2A regulatory subunit beta isoform</td><td colspan="2">ns</td><td>-1,29</td><td>0.042</td></tr>
<tr><td>PPP2R5A</td><td>Serine/threonine-protein phosphatase 2A regulatory subunit alpha isoform</td><td colspan="2">ns</td><td>-1,18</td><td>0.035</td></tr>
<tr><td>PPP2R5D</td><td>Serine/threonine-protein phosphatase 2A regulatory subunit delta isoform</td><td colspan="2">ns</td><td>-1,37</td><td>< 0.001</td></tr>
<tr><td>PPP2R3A</td><td>Serine/threonine-protein phosphatase 2A regulatory subunit B'' subunit alpha</td><td colspan="2">ns</td><td>-1,23</td><td>0.004</td></tr>
<tr><td>PPP2R2D</td><td>Serine/threonine-protein phosphatase 2A regulatory subunit B delta isoform</td><td>-1,48</td><td>< 0.001</td><td>-1,41</td><td>< 0.001</td></tr>
<tr><td>PPP2CB</td><td>Serine/threonine-protein phosphatase 2A catalytic subunit beta isoform</td><td>-1,18</td><td>< 0.001</td><td>-1,33</td><td>< 0.001</td></tr>
</table>

Supplementary Table S3. Primer sequences and probes used for quantitative reverse transcription-PCR (qRT-PCR).

Name	Fw/Rv	Sequence	Probe (UPL, Roche)
Tenascin C	Fw	5'-CCGGACCAAAACCATCAGT-3'	76
	Rv	5'-GGGATTAATGTCGGAAATGGT-3'	
Lumican	Fw	5'-GAAAGCAGTGTCAAGACAGTAAGG-3'	72
	Rv	5'-GGCCACTGGTACCACCAA-3'	
CD24	Fw	5'-TGGATTTGACATTGCATTTGA-3'	37
	Rv	5'-TGGGGGTAGATTCTCATTCATC-3'	
COL4A3	Fw	5'-CTACGGACCCCAAGGAGAA-3'	1
	Rv	5'-GGCCTGGAACTGGTGTTG-3'	
COL4A4	Fw	5'-TGGTCCTCCAGGTCCAAA-3'	27
	Rv	5'-CTCTTTCTCCGGGAAAACCT-3'	
COL6A1	Fw	5'-GAAGAGAAGGCGCCGTTG-3'	80
	Rv	5'-CGGTAGCCTTTAGGTCCGATA-3'	
COL6A2	Fw	5'-AGACCTTCCCTGCCAAACA-3'	4
	Rv	5'-CTTGTGGAAGTTCTGCTCACC-3'	
COL6A3	Fw	5'-TCTTTTGCCTCTTTCTCTCAGG-3'	3
	Rv	5'-CTCTTGTTGTTGACTTCAATGACTGC-3'	
SMAD1	Fw	5'-TCAGCCGATGGACACAAAC-3'	82
	Rv	5'-TCATAAGCAACCGCCTGAA-3'	
SMAD5	Fw	5'-AACCCCTCATTCATCTTAGCC-3'	50
	Rv	5'-CCAACTTCCCTTTGAAAAAGC-3'	
SALL4	Fw	5'-ACTGCAACCTCCCCATTCT-3'	2
	Rv	5'-CCCAGCTACTCTGGAGGCTA-3'	
NR5A2	Fw	5'-TTTGGCATTGTTGGATTTCA-3'	4
	Rv	5'-TTGGATGATGAAAAAGACTGATG-3'	
PECAM1	Fw	5'-GCTTCCCGATAAGATCTCAGG-3'	1
	Rv	5'-TCCCAGAGGAAGGTAGGTCAT-3'	
MAP4K4	Fw	5'-CACTTGGAAGTCCTTCAGCA-3'	1
	Rv	5'-GCCTCCTATGGTCATGCAGT-3'	
GLI2	Fw	5'-AAGGAGAGGGGACTGTTTGG-3'	1
	Rv	5'-ACGAGGGTCATCTGGTGGT-3'	
B3GNT7	Fw	5'-CCTGAGCCCCCATGGTAT-3'	19
	Rv	5'-TGGGGACCAAAGGCTAGG-3'	
LEF1	Fw	5'-GCGGAGCGGAGATTACAG-3'	10
	Rv	5'-TTGAAGGGGATCATCTCGTC-3'	
TXNIP	Fw	5'-GGCTAAAGTGCTTTGGATGC-3'	3
	Rv	5'-TGTCTTCATAGCGCAGGTACTC-3'	
HAS2	Fw	5'-GCCACCAAAGGAGACATATCA-3'	3
	Rv	5'-GGCAGAATGAAAATAAACCCATA-3'	
DUSP1	Fw	5'-CGAGGCCATTGACTTCATAGA-3'	65
	Rv	5'-CTGGCAGTGGACAAACACC-3'	
DUSP5	Fw	5'-CCTACAGGGTTTGTAGGGACAT-3'	89
	Rv	5'-TCTACACAACATTTCAGGGTTCA-3'	
DUSP8	Fw	5'-CCAGTCACTGTGGGAAGAGG-3'	1
	Rv	5'-ACAAATAGAAGAAACAGCAGAGAGG-3'	
DUSP10	Fw	5'-TCCCTCCAGCTCCAAGAGT-3'	10
	Rv	5'-ATGGGCTGAGGTAGCAAGC-3'	
DUSP12	Fw	5'-AACCCCTCATTCATCTTAGCC-3'	50
	Rv	5'-CCAACTTCCCTTTGAAAAAGC-3'	
DUSP14	Fw	5'-GTGGCTGACAAGATCCACAG-3'	4
	Rv	5'-GCAGCACAGTGCACCAAG-3'	
DUSP16	Fw	5'-AGCCGCACAGACCTCACTA-3'	8
	Rv	5'-CATAACAATGCGGCTGACC-3'	
PPP2CA	Fw	5'-TTGTTGGTGTGCACTTTGTG-3'	4
	Rv	5'-TGAAGCAGCACAGATTTCCTT-3'	
PPP2CB	Fw	5'-GGACGACAAGGCGTTCAC-3'	17
	Rv	5'-TCAGCTGCTTACACTCGTTCA-3'	
PPP2R1B	Fw	5'-TGGATATGAAACTGTAGAGGGAGA-3'	2
	Rv	5'-TGCAGTCACCTCAGCTGCTA-3'	
PPP2R2A	Fw	5'-GCAACAGGAGATAAAGGTGGTAG-3'	2
	Rv	5'-TCCTCTGCTATGAGACTGGATTT-3'	
PPP2R5B	Fw	5'-CGCAAACAGTGCAACCAC-3'	69
	Rv	5'-ACACCATTGAAGTGCTCGAA-3'	
PPP2R5A	Fw	5'-TCTGGAATTTAGGCAAAACCTT-3'	80
	Rv	5'-CCCAGCTTTTATTTATATTTTGTGC-3'	
PPP2R5D	Fw	5'-CGCTTCAACCTCAGCAAGA-3'	4
	Rv	5'-GGGTTGGCGAATCTTTCA-3'	
PPP2R3A	Fw	5'-ACCCAGGGTCAAGAGAGGAT-3'	2
	Rv	5'-TTCCCACTGGCAAAACTGAT-3'	
PPP2R2D	Fw	5'-TGTGTGCAGCATTTCTGTCC-3'	74
	Rv	5'-CCTGGTGTATCTGATGGGCTA-3'	
18S	Fw	5'-CTTCCACAGGAGGCCTACAC-3'	48
	Rv	5'-CGCAAAATATGCTGGAACTTT-3'	

Supplementary Table S4. Antibodies and dilution used for Western Blot experiments.

Protein targeted	Source	Clone/Cat#	Dilution (WB)
**Argpyrimidine**	Oya et al. JBC 1999	mAb6B	1/6000
**β-actin**	Sigma-Aldrich (St Louis, MO, USA)	A5441	1/5000
**E-cadherin**	BD Biosciences (Franklin Lakes, NJ, USA)	610181	1/1000
**Collagen VI α1**	Lifespan Biosciences (Seattle, WA, USA)	LSB696	1/1000
**Collagen VI α3**	Sigma-Aldrich (St Louis, MO, USA)	SAB4500389	1/1000
**Tenascin C**	ThermoFischer Scientific (Waltham, MA, USA)	MA1-26779	1/1000
**TGFBR1**	Santa Cruz (Santa Cruz, CA, USA)	sc-398	1/1000
**Glyoxalase 1**	BioMAC (Leipzig, Germany)	#02-14	1/1000
**SMAD4**	Cell Signaling (Danvers, MA, USA)	#38454	1/1000
**P-MEK1/2**	Cell Signaling (Danvers, MA, USA)	#2338	1/1000
**MEK2**	Cell Signaling (Danvers, MA, USA)	#9125	1/1000
**P-ERK1/2**	Cell Signaling (Danvers, MA, USA)	#9101	1/1000
**ERK1/2**	Cell Signaling (Danvers, MA, USA)	#9102	1/1000
**MG-H (3D11)**	Cell Biolabs (San Diego, CA, USA)	STA-011	1/2000
**Nrf2**	Abcam (Cambridge, UK)	ab62352	1/1000
**TGFBI**	Cell Signaling (Danvers, MA, USA)	#5601	1/1000
**Phospho-Smad2 (Ser465/467)/Smad3 (Ser423/425)**	Cell Signaling (Danvers, MA, USA)	#8828	1/500
**SMAD2/3**	Cell Signaling (Danvers, MA, USA)	#8685	1/1000
**P-SMAD1 (ser206)**	Cell Signaling (Danvers, MA, USA)	#5753	1/1000
**P-SMAD1/5 (ser463/465)**	Cell Signaling (Danvers, MA, USA)	#9516	1/1000
**SMAD1**	Cell Signaling (Danvers, MA, USA)	#6944	1/1000
**Vimentin**	Sigma-Aldrich (St Louis, MO, USA)	V6389	1/1000
**SMAD5**	Cell Signaling (Danvers, MA, USA)	#12534	1/1000
**DUSP1**	Abcam (Cambridge, UK)	ab195261	1/1000
**DUSP5**	Santa Cruz (Santa Cruz, CA, USA)	sc-393801	1/1000
**DUSP8**	Santa Cruz (Santa Cruz, CA, USA)	sc-271250	1/1000
